# SARS-CoV-2 infection causes dopaminergic neuron senescence

**DOI:** 10.1016/j.stem.2023.12.012

**Published:** 2024-02-01

**Authors:** Liuliu Yang, Tae Wan Kim, Yuling Han, Manoj S. Nair, Oliver Harschnitz, Jiajun Zhu, Pengfei Wang, So Yeon Koo, Lauretta A. Lacko, Vasuretha Chandar, Yaron Bram, Tuo Zhang, Wei Zhang, Feng He, Chendong Pan, Junjie Wu, Yaoxing Huang, Todd Evans, Paul van der Valk, Maarten J. Titulaer, Jochem K.H. Spoor, Robert L. Furler O’Brien, Marianna Bugiani, Wilma D.J. Van de Berg, Robert E. Schwartz, David D. Ho, Lorenz Studer, Shuibing Chen

**Affiliations:** 1 Department of Surgery, Weill Cornell Medicine, 1300 York Ave., New York, NY 10065, USA; 2 Center for Genomic Health, Weill Cornell Medicine, 1300 York Ave., New York, NY 10065, USA; 3 The Center for Stem Cell Biology, Sloan-Kettering Institute for Cancer Research, New York, NY 10065, USA; 4 Developmental Biology Program, Sloan-Kettering Institute for Cancer Research, New York, NY 10065, USA; 5 Aligning Science Across Parkinson's (ASAP) Collaborative Research Network, Chevy Chase, MD 20815, USA; 6 Aaron Diamond AIDS Research Center, Columbia University Vagelos College of Physicians and Surgeons, New York, NY 10032, USA; 7 Human Technopole, Viale Rita Levi-Montalcini, 1, 20157 Milan, Italy; 8 Neuroscience Graduate Program of Weill Cornell Graduate School of Biomedical Sciences, New York, NY, USA; 9 Division of Gastroenterology and Hepatology, Department of Medicine, Weill Cornell Medicine, 1300 York Ave., New York, NY 10065, USA; 10 Department of Physiology, Biophysics and Systems Biology, Weill Cornell Medicine, 1300 York Ave., New York, NY 10065, USA; 11 Genomic Resource Core Facility, Weill Cornell Medicine, New York, NY 10065, USA; 12 Department of Pathology, Amsterdam University Medical Center, VU University Amsterdam, Amsterdam, the Netherlands; 13 Department of Neurology, Erasmus University Medical Center, Rotterdam, the Netherlands; 14 Department of Neurosurgery, Erasmus University Medical Center, Rotterdam, the Netherlands; 15 Division of Infectious Diseases, Department of Medicine, Weill Cornell Medicine, 1300 York Ave., New York, NY 10065, USA; 16 Amsterdam UMC, Location Vrije Universiteit Amsterdam, Department of Pathology, De Boelelaan 1117, Amsterdam, the Netherlands; 17 Amsterdam Neuroscience, Neurodegeneration, Amsterdam, the Netherlands; 18 Amsterdam UMC, Location Vrije Universiteit Amsterdam, Department of Anatomy and Neurosciences, Section Clinical Neuroanatomy and Biobanking, De Boelelaan 1117, Amsterdam, the Netherlands

**Keywords:** human pluripotent stem cells, SARS-CoV-2, dopaminergic neuron, senescence

## Abstract

COVID-19 patients commonly present with signs of central nervous system and/or peripheral nervous system dysfunction. Here, we show that midbrain dopamine (DA) neurons derived from human pluripotent stem cells (hPSCs) are selectively susceptible and permissive to severe acute respiratory syndrome coronavirus 2 (SARS-CoV-2) infection. SARS-CoV-2 infection of DA neurons triggers an inflammatory and cellular senescence response. High-throughput screening in hPSC-derived DA neurons identified several FDA-approved drugs that can rescue the cellular senescence phenotype by preventing SARS-CoV-2 infection. We also identified the inflammatory and cellular senescence signature and low levels of SARS-CoV-2 transcripts in human substantia nigra tissue of COVID-19 patients. Furthermore, we observed reduced numbers of neuromelanin+ and tyrosine-hydroxylase (TH)+ DA neurons and fibers in a cohort of severe COVID-19 patients. Our findings demonstrate that hPSC-derived DA neurons are susceptible to SARS-CoV-2, identify candidate neuroprotective drugs for COVID-19 patients, and suggest the need for careful, long-term monitoring of neurological problems in COVID-19 patients.

## Highlights

• hPSC-derived DA neurons are susceptible to SARS-CoV-2 infection
• SARS-CoV-2 infection of DA neurons triggers cellular senescence response
• Several FDA-approved drugs were identified to rescue senescence of DA neurons
• Cellular senescence was found in substantia nigra tissues of COVID-19 patients

## Graphical abstract

**Figure:**
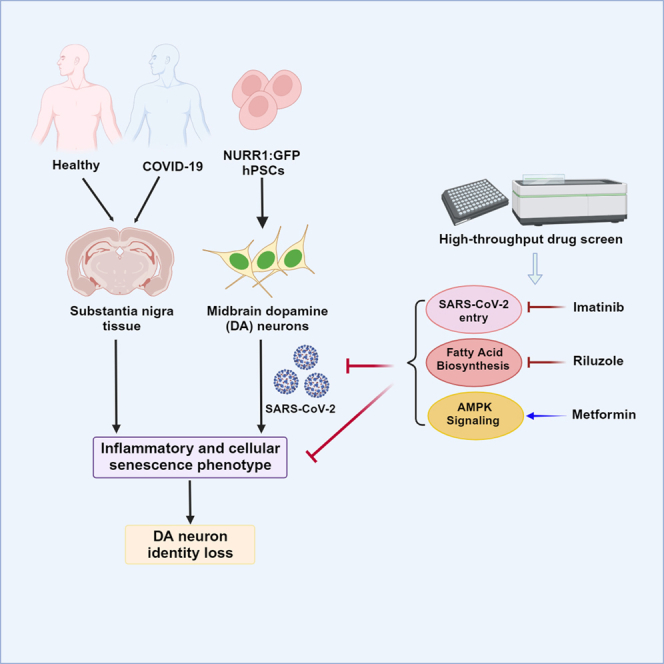

## Teaser

Yang and colleagues demonstrate that SARS-CoV-2 can infect hPSC-derived midbrain dopamine (DA) neurons and induce cellular senescence. Several drug candidates were identified to rescue SARS-CoV-2 infection and DA neuron senescence. Inflammation and cellular senescence were also identified in substantia nigra tissue of COVID-19 patients.

## Introduction

Abnormal neurological manifestations are increasingly recognized in patients with COVID-19, which most commonly include anosmia, dysgeusia, and headache, followed by seizures, stroke, and acute inflammatory polyradiculoneuropathy, also known as Guillain-Barre syndrome.^1^ Furthermore, an increased risk for additional neurological and psychiatric disorders has been reported in a large retrospective cohort at 6 months post diagnosis.^2^ Recent studies using hPSC-derived organoid models have established that choroid plexus cells within the CNS are highly susceptible to severe acute respiratory syndrome coronavirus 2 (SARS-CoV-2) infection.^3^^,^^4^ However, the tropism of SARS-CoV-2 for neurons has remained controversial.^3^^,^^4^^,^^5^^,^^6^ We previously developed a human pluripotent stem cell (hPSC)-derived organoid/cell-based platform to evaluate the tropism of SARS-CoV-2. Using this platform, we found that hPSC-derived midbrain dopamine (DA) neurons—representing one of the main neurodegeneration targets in Parkinson’s disease (PD)—are permissive to SARS-CoV-2 infection.^7^ Conversely, under identical experimental conditions, we found that hPSC-derived cortical neurons are not permissive to SARS-CoV-2 infection,^7^ supporting the notion that not all neuronal populations are equally permissive to viral infection. Here, we set out to define how DA neurons respond to SARS-CoV-2 infection and to determine the molecular changes induced by SARS-CoV-2 infection.

## Results

### hPSC-derived DA neurons are susceptible and permissive to SARS-CoV-2 infection

To examine the impact of SARS-CoV-2 infection on DA neurons, postmitotic DA neuron marker (NURR1: GFP), reporter hPSCs were differentiated toward a DA neuron fate using a previously established strategy.^8^^,^^9^ Our previous work found that hPSC-derived midbrain DA neurons are susceptible to a vesicular stomatitis ΔG-luciferase virus pseudotyped with the SARS-CoV-2 Spike protein incorporated at the surface of the viral particle (SARS-CoV-2-entry virus).^7^ To focus our study on purified DA neurons in culture, we sorted NURR1-GFP^+^ cells at day 25 of differentiation. In this study, DA neuron identity was validated by immunofluorescent staining with NURR1-GFP, tyrosine-hydroxylase (TH), MAP2, and FOXA2 showing co-expression of TH and FOXA2 at day 40 (Figures S1A and S1B). We used those highly purified populations of DA neurons, resulting in up to 90% of the NURR1^+^ cells exhibiting DA neuron identity, consistent with the purity reported in our previous single-cell real-time quantitative PCR study (Figure S1C),^8^ to monitor their response to SARS-CoV-2 infection.

First, we validated the expression of ACE2, the SARS-CoV-2 receptor, in NURR1: GFP^+^ DA neurons by immunostaining (Figures S1D and S1E). Then, DA neuron susceptibility to SARS-CoV-2 entry was further confirmed using the SARS-CoV-2-entry virus^10^^,^^11^ resulting in robust luciferase activity in infected DA neurons (Figure S1F). Immunostaining demonstrated that around 5% of NURR1:GFP^+^ cells were positively stained by luciferase antibody (Figures S1G and S1H).

**Figure 1:**
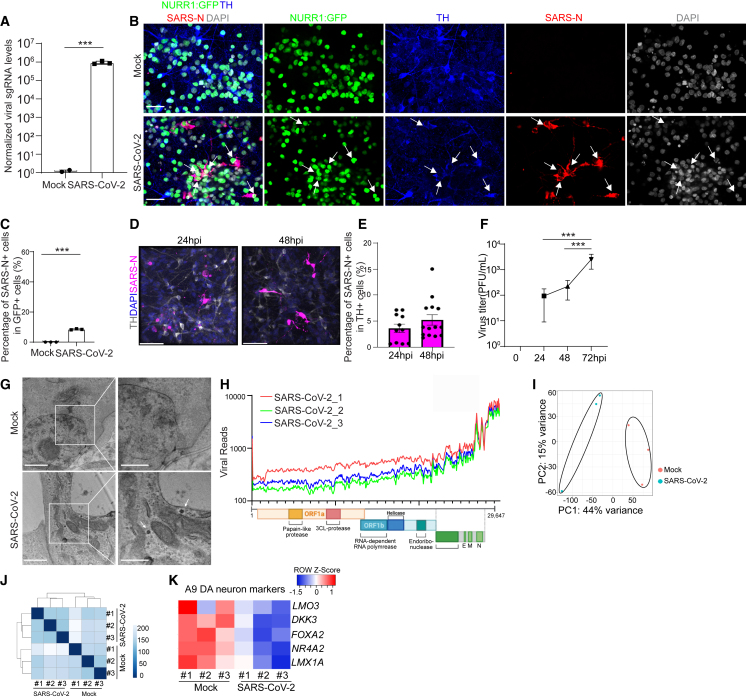
hPSC-derived DA neurons are susceptible and permissive to SARS-CoV-2 infection (A) Real-time quantitative PCR analysis of total RNA from purified NURR1:GFP H9-derived DA neurons at 48 hpi of SARS-CoV-2 infection (MOI = 0.2) for viral N sgRNA (small guide RNA). The graph depicts the mean sgRNA level normalized to *ACTB*. n = 3 independent biological replicates. (B and C) Representative confocal images (B) and quantification (C) of purified NURR1:GFP H9-DA neurons infected with SARS-CoV-2 (MOI = 0.1) at 72 hpi using antibodies against SARS-CoV-2 nucleocapsid protein (SARS-N) and markers for DA neurons. Scale bars, 50 μm. n = 3 independent biological replicates. (D and E) Immunostaining (D) and quantification (E) of *SARS-N* in SARS-CoV-2 infected purified NURR1:GFP H9-derived DA neurons at 24 or 48 hpi (MOI = 0.2). n = 3 independent biological replicates. (F) Virus endpoint titration assay from supernatants of purified NURR1:GFP H9-DA neurons infected with SARS-CoV-2 (MOI = 0.2) at different time points. n = 3 independent biological replicates. (G) Transmission electron microscopy (TEM) images of purified NURR1:GFP H9-DA neurons at 72 hpi of SARS-CoV-2 (MOI = 1.0). Arrows point to SARS-CoV-2 viral particles. Right panel: zoom in images. Scale bars, 1 μm. n = 3 independent biological replicates. (H) RNA-seq read coverage of the viral genome in purified NURR1:GFP H9-DA neurons at 48 hpi (MOI = 0.2). The schematic below depicts the SARS-CoV-2 genome. (I) PCA plot of gene expression profiles from mock or SARS-CoV-2 infected purified NURR1:GFP H9-DA neurons at 48 hpi (MOI = 0.2). (J) Clustering analysis of mock or SARS-CoV-2 infected purified NURR1:GFP-DA neurons at 48 hpi (MOI = 0.2). (K) Heatmap of expression levels of DA neurons and A9 DA neuron marker genes in the mock or SARS-CoV-2 infected purified NURR1:GFP H9-DA neurons at 48 hpi (MOI = 0.2). Data were presented as mean ± SD. p values were calculated by unpaired two-tailed Student’s t test. ^∗∗∗^p < 0.001. See also Figures S1 and S3.

Next, hPSC-derived purified DA neurons were infected *in vitro* with SARS-CoV-2 (USA-WA1/2020, multiplicity of infection [MOI] = 0.2). At 48 h post infection (hpi), real-time quantitative PCR analysis using primers targeting subgenomic *N* transcripts detected significant amounts of viral replication at the RNA level in infected hPSC-derived DA neurons (Figure 1A). Immunostaining for SARS-CoV-2 nucleocapsid protein (SARS-N) antibody confirmed robust SARS-CoV-2 infection in approximately 8% of NURR1:GFP^+^ DA neurons at 72 hpi (Figures 1B and 1C). Early time points showed a trend toward increased SARS-CoV-2 infection to TH^+^ DA neurons from 24 to 48 hpi (Figures 1D and 1E). Moreover, endpoint titer determining assays confirmed productive infection in hPSC-derived DA neurons with the robust generation of infectious virus at 24, 48, and 72 hpi (Figure 1F). Furthermore, transmission electron microscopy (TEM) detected the presence of viral particles in SARS-CoV-2 infected hPSC-derived purified DA neurons (Figure 1G). In addition, we tested both low MOI (MOI = 0.2) and high MOI (MOI = 1) infection of DA neurons and detected higher percentage of SARS-N^+^ cells at 72 hpi of high MOI condition than that at low MOI condition (Figures S1I and S1J). ACE2 blocking antibody prevented virus infection at both low and high MOI conditions (Figures S1I and S1J), suggesting that SARS-CoV-2 infection of hPSC-derived DA neurons is dependent on ACE2 receptor interactions. Since the percentage of SARS-CoV-2 infection in DA neurons is not very high, we cannot fully exclude the possibility that the initially infected DA neurons can produce interferon, which protects the non-infected DA neurons.

RNA sequencing (RNA-seq) analysis was then applied to compare mock-infected or SARS-CoV-2 infected hPSC-derived purified DA neurons. Robust viral infection was detected in SARS-CoV-2 infected DA neurons (Figure 1H). Moreover, plotting these datasets by principal-component analysis (PCA, Figure 1I) and clustering analysis (Figure 1J) demonstrated that the infected DA neurons occupied a distinct transcriptional space compared with mock-infected DA neurons. Interestingly, the expression of midbrain DA neuron markers such as *NR4A2* (encoding protein NURR1), *FOXA2*, and *LMX1A*, and in particular, A9 (substantia nigra) DA neuron subtype-enriched markers *DKK3* and *LMO3*^12^, were decreased in SARS-CoV-2 infected samples (Figure 1K).

**Figure 2:**
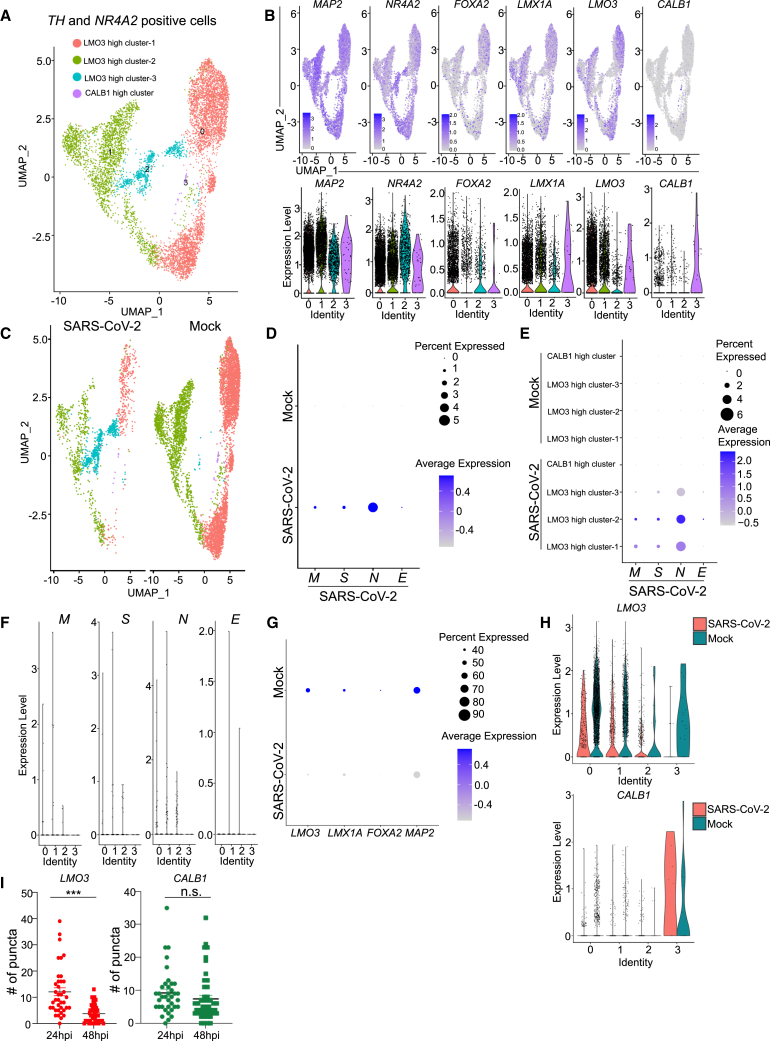
hPSC-derived *TH* and *NR4A2* positive DA neurons are susceptible and permissive to SARS-CoV-2 infection (A) Uniform Manifold Approximation and Projection (UMAP) analysis of mock and SARS-CoV-2 infected purified NURR1:GFP H9-derived *TH* and *NR4A2* positive DA neurons at 48 hpi (MOI = 0.2). (B) UMAP and violin plot analysis of DA neuron marker genes. (C) UMAP analysis of mock and SARS-CoV-2 infected NURR1:GFP H9-derived *TH* and *NR4A2* positive DA neurons at 48 hpi (MOI = 0.2). (D) Dot plot analysis of SARS-CoV-2 viral transcripts in mock and SARS-CoV-2 infected NURR1:GFP H9-derived *TH* and *NR4A2* positive DA neurons at 48 hpi (MOI = 0.2). (E) Dot plot analysis of SARS-CoV-2 viral transcripts in different populations of mock and SARS-CoV-2 infected NURR1:GFP H9-derived *TH* and *NR4A2* positive DA neurons at 48 hpi (MOI = 0.2). (F) Violin plot analysis of SARS-CoV-2 viral transcripts in different populations of mock and SARS-CoV-2 infected NURR1:GFP H9-derived *TH* and *NR4A2* positive DA neurons at 48 hpi (MOI = 0.2). (G) Dot plot analysis of DA neuron marker genes in mock and SARS-CoV-2 infected NURR1:GFP H9-derived *TH* and *NR4A2* positive DA neurons at 48 hpi (MOI = 0.2). (H) Violin plot analysis of DA neuron marker genes in different populations of mock and SARS-CoV-2 infected NURR1:GFP H9-derived *TH* and *NR4A2* positive DA neurons at 48 hpi (MOI = 0.2). (I) Fluorescence *in situ* hybridization and quantification of A9 DA neuron subtype marker, *LMO3*, and A10 DA neuron subtype marker, *CALB1* in mock or SARS-CoV-2 infected purified NURR1:GFP H9-DA neurons at 48 hpi (MOI = 0.2). n = 3 independent biological replicates. Data were presented as mean ± SD. p values were calculated by unpaired two-tailed Student’s t test. ^∗∗∗^p < 0.001. n.s., no significance. See also Figure S2.

To study DA subtype-specific vulnerability to SARS-CoV-2 infection, we performed scRNA-seq from hPSC-derived purified DA neurons at day 40 in mock or SARS-CoV-2 infected (MOI = 0.2) neurons. Although we ran the scRNA-seq from GFP^+^ purified cells corresponding to NR4A2 expression, due to the detection sensitivity^13^^,^^14^ and decreased expression of *NR4A2* upon SARS-CoV-2 infection, we were not able to detect *NR4A2* in all cells. To further confirm the infection of DA neurons, we analyzed both *TH* and *NR4A2* positive cells as well as all cells (Figures 2 and S2). Clustering analysis identified four cell populations expressing DA neuron markers, including *MAP2*, *NR4A2*, and *LMX1A* (Figures 2B and S2B), and we mark those clusters as *LMO3* high cluster-1, *LMO3* high cluster-2, *LMO3* high cluster-3, and *CALB1* high cluster (Figures 2A–2C and S2A–S2C). One cluster shows high expression of *CALB1*, an A10 DA neuron marker,^15^^,^^16^ whereas the other three clusters expressed higher level of *LMO3*, which is considered an A9-DA neuron marker^12^ (Figures 2B and S2B). There is no other cell population detected based on scRNA-seq analysis. Together, it suggests hPSC-derived NURR1-GFP-sorted neurons are a relatively pure DA neuron population, mainly consisting of A9-like DA neurons, the subtype of substantia nigra DA neurons most affected in PD.^12^

SARS-CoV-2 viral transcripts, including *SARS-CoV-2-M*, *SARS-CoV-2-N*, *SARS-CoV-2-S*, and *SARS-CoV-2-E*, are highly detected in SARS-CoV-2 infected cells (Figures 2D and S2D) in particular *LMO3* high cluster-1, *LMO3* high cluster-2, and *LMO3* high cluster-3. By contrast, those viral transcripts are absent in the *CALB1* high cluster (Figures 2E and 2F). Dot plots showed the decrease of A9 DA neuron marker expression (Figures 2G, 2H, S2E, and S2F), which is consistent with the bulk RNA-seq results (Figure 1K). Finally, quantitative RNA *in situ* hybridization further confirmed the decrease of A9 marker *LMO3*, but not A10 marker *CALB1* expression following SARS-CoV-2 infection, indicating the loss of cell identity of A9-type DA neurons upon SARS-CoV-2 infection (Figure 2I).

To further validate the susceptibility and permissiveness of hPSC-derived DA neurons to SARS-CoV-2 infection and replication, we derived DA neurons from one additional human embryonic stem cell (hESC) line (MEL-1) and one PD-control induced PSC (iPSC) line (2 copy of SNCA DNA,^17^ equivalent to healthy control). Real-time quantitative PCR and immunostaining confirmed the robust SARS-CoV-2 infection of DA neurons derived from MEL-1 hESCs (Figures S3A–S3C) and PD-control iPSCs (Figures S3D and S3E). Together, these experiments confirm that hPSC-derived DA neurons are susceptible to SARS-CoV-2 and support productive infection.

Finally, we also examined the impact of SARS-CoV-2 infection on hPSC-derived cortical neurons (Figure S3F). Consistent with our previous reports using SARS-CoV-2 entry virus,^7^ there are few SARS-N positive cells detected in hPSC-derived cortical neurons at 72 hpi at MOI = 0.2 (Figure S3G). Besides, no obvious transcriptional changes were observed following SARS-CoV-2 exposure of hPSC-derived cortical neurons (Figure S3H).

### SARS-CoV-2 infection induces senescence of DA neurons

**Figure 3:**
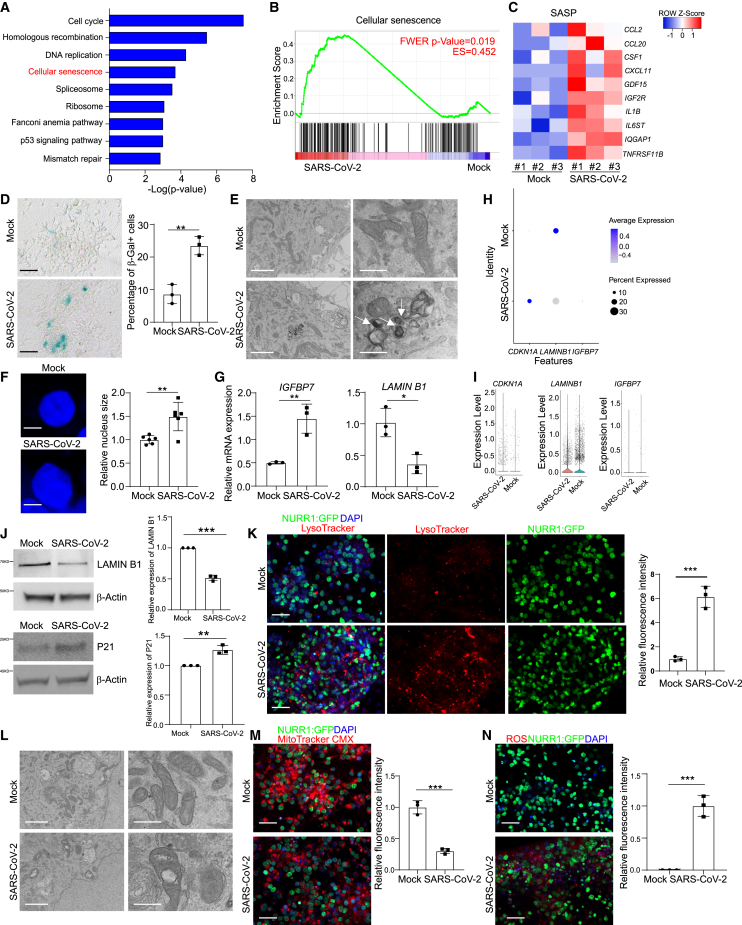
SARS-CoV-2 infection induces senescence of DA neurons (A) Ingenuity pathway analysis (IPA) of differentially expressed genes between mock or SARS-CoV-2 infected purified NURR1:GFP H9-DA neurons at 48 hpi (MOI = 0.2). (B) Gene set enrichment analysis (GSEA) of cellular senescence pathway in mock or SARS-CoV-2 infected purified NURR1:GFP H9-DA neurons at 48 hpi (MOI = 0.2). (C) Heatmap of SASP associated genes in mock or SARS-CoV-2 infected purified NURR1:GFP H9-DA neurons at 48 hpi (MOI = 0.2). (D) β-gal staining (left) and quantification (right) of the percentage of β-Gal^+^ cells of mock or SARS-CoV-2 infected purified NURR1:GFP H9-DA neurons at 72 hpi (MOI = 0.1). Scale bars, 75 μm. n = 3 independent biological replicates. (E) TEM images of mock or SARS-CoV-2 infected purified NURR1:GFP H9-DA neurons at 72 hpi (MOI = 1.0). Scale bars, 2 μm. n = 3 independent biological replicates. (F) Immunostaining of DAPI (left) and quantification (right) of relative nuclear size in mock or SARS-CoV-2 infected purified NURR1:GFP H9-DA neurons at 72 hpi (MOI = 0.1). Scale bars, 75 μm. n = 3 independent biological replicates. (G) Real-time quantitative PCR analysis of *IGFBP7* and *LAMIN B1* in mock or SARS-CoV-2 infected purified NURR1:GFP H9-DA neurons at 48 hpi (MOI = 0.2). n = 3 independent biological replicates. (H) Dot plot analysis of *CDKN1A*, *IGFBP7*, and *LAMIN B1* in mock or SARS-CoV-2 infected purified NURR1:GFP H9-DA neurons at 48 hpi (MOI = 0.2). (I) Violin plot analysis of *CDKN1A*, *IGFBP7*, and *LAMIN B1* in mock or SARS-CoV-2 infected purified NURR1:GFP H9-DA neurons at 48 hpi (MOI = 0.2). (J) Western blot analysis (left) and quantification (right) of P21 and LAMIN B1 in mock or SARS-CoV-2 infected purified NURR1:GFP H9-DA neurons at 48 hpi (MOI = 0.2). n = 3 independent biological replicates. (K) Representative confocal images (left) and quantification (right) of relative LysoTracker intensity of purified NURR1:GFP H9-DA neurons infected with mock or SARS-CoV-2 (MOI = 0.1) at 72 hpi. Scale bars, 50 μm. n = 3 independent biological replicates. (L) Representative TEM images of mitochondrial in purified NURR1:GFP H9-DA neurons infected with mock or SARS-CoV-2 (MOI = 0.1) at 72 hpi. Scale bars, 50 μm. n = 3 independent biological replicates. (M) Representative confocal images (left) and quantification (right) of MitoTracker intensity of purified NURR1:GFP H9-DA neurons infected with mock or SARS-CoV-2 (MOI = 0.1) at 72 hpi. Scale bars, 50 μm. n = 3 independent biological replicates. (N) Representative confocal images (left) and quantification (right) of ROS intensity of purified NURR1:GFP H9-DA neurons infected with mock or SARS-CoV-2 (MOI = 0.1) at 72 hpi. Scale bars, 50 μm. n = 3 independent biological replicates. Data were presented as mean ± SD. p values were calculated by unpaired two-tailed Student’s t test. ^∗∗^p < 0.01, and ^∗∗∗^p < 0.001. See also Figures S3 and S4.

Ingenuity pathway analysis of genes differentially expressed between mock and SARS-CoV-2 infected hPSC-derived DA neurons highlighted the cell cycle, DNA replication, and senescence pathways as the top upregulated pathways in SARS-CoV-2 infected DA neurons (Figure 3A). Gene set enrichment analysis (GSEA) further confirmed the enrichment of senescence pathway in SARS-CoV-2 versus mock-infected purified hPSC-derived DA neurons (Figure 3B). Further analysis of RNA-seq data of purified hPSC-derived DA neurons using NURR1-GFP in mock-infected or SARS-CoV-2-infected conditions identified robust induction of chemokine/cytokine transcripts and inflammation-related genes (Figures S3I and S3J). Senescence-associated genes are also significantly upregulated in SARS-CoV-2 infected DA neurons (Figure S3K). In stark contrast, the senescence pathway was not significantly enriched in SARS-CoV-2 infected lung organoids, pancreatic cells, liver organoids, and cardiomyocytes (Figure S3L).

A key feature of senescent cells is the activation of the senescence-associated secretory phenotype (SASP). There was an upregulation of SASP associated genes, including *CCL2*, *CCL20*, *CSF1*, *CXCL11*, *GDF15*, *IGF2R*, *IL1B*, *IL6ST*, *IQGAP1*, and *TNFRSF11B* upon SARS-CoV-2 infection in DA neurons (Figure 3C). Acidic lysosomal senescence-associated β-galactosidase (SA-β-gal) activity, a biomarker of cellular senescence,^8^ was also upregulated in SARS-CoV-2 infected hPSC-derived DA neurons (Figure 3D). Transmission EM detected lipofuscin in SARS-CoV-2 infected DA neurons, another senescence-associated marker of DA neurons^18^ (Figure 3E). In addition, SARS-CoV-2 infected DA neurons also showed increased nuclear size (Figure 3F). Real-time quantitative PCR analysis showed the upregulation of *IGFBP7* and downregulation of *LMNB1*, genes reported to be associated with senescence in DA neurons^8^^,^^19^ (Figure 3G). scRNA-seq analysis confirmed the upregulation of *IGFBP7* and *CDKN1A* (encoding protein P21) and downregulation of *LMNB1* (Figures 3H and 3I). Western blotting further validated the upregulation of P21 and downregulation of LMNB1 at the protein level (Figure 3J). Moreover, SARS-CoV-2 infected DA neurons showed additional senescence-associated phenotypes, including increased accumulation of lysosomes as indicated by LysoTracker staining (Figure 3K), mitochondrial dysfunction as indicated by EM and MitoTracker staining (Figures 3L and 3M), and increased protein oxidation as indicated by ROS staining (Figure 3N). Consistent with the data of H9 hESC-derived DA neurons, MEL-1 hESC-derived DA neurons also showed increased β-gal staining and lysosomal accumulation upon SARS-CoV-2 infection (Figures S3M and S3N). By contrast, in hPSC-derived cortical neurons, SARS-CoV-2 infection neither increased β-gal staining nor upregulated SASP associated genes, which is consistent with our previous data showing a lack of susceptibility of cortical neurons to SARS-CoV-2^7^ (Figures S3O and S3P).

Since DA neurons are one of the major cell types degenerated in PD, and DA neuron senescence has been shown to be associated with PD,^20^ we next monitored how PD-iPSC-derived DA neurons responded to SARS-CoV-2 infection. *SNCA*, the first gene associated with familial PD, encodes the protein α-synuclein (α-Syn).^21^ Copy-number variations (CNVs) in the *SNCA* gene have been identified in patients with familial PD,^22^^,^^23^ and SNPs in *SNCA* are also strongly associated with sporadic PD.^24^ Here, we used isogenic *SNCA*^17^ 4 copy, 2 copy, and 0 copy iPSC-derived DA neurons to determine their response to SARS-CoV-2 infection. First, *SNCA* copy numbers do not affect the differentiation toward MAP2^+^FOXA2^+^LMX1^+^TH^+^ DA neurons (Figure S4A). Immunostaining showed that *SNCA* 4 copy iPSC-derived DA neurons displayed higher total α-Syn expression compared with *SNCA* 2 copy iPSC-derived DA neurons, whereas *SNCA* 0 copy iPSC-derived DA neurons showed no expression when using an antibody against total α-Syn (Figure S4B). Interestingly, real-time quantitative PCR analysis detected increased levels of SARS-CoV-2 subgenomic *N* RNA in SNCA 4 copy iPSC-derived DA neurons compared with SNCA 2 copy and SNCA 0 copy iPSC-derived DA neurons at 48 hpi (Figure S4C). Although the mechanism of increased SARS-CoV2 susceptibility in *SNCA* 4 copy DA neurons remains to be determined, previous studies have shown that the SARS-CoV-2 S protein can bind to TLR4, which is upregulated in PD,^25^ expressed on the neuron surface, and which facilitates the viral entry.^26^^,^^27^

Next, we assessed senescence and disease associated phenotypes in mock and SARS-CoV-2-infected DA neurons derived from isogenic *SNCA* 4 copy, 2 copy, and 0 copy hiPSC lines. SARS-CoV-2 infection increased both the percentage of β-gal^+^ cells (Figure S4D) and lysosomal accumulation (Figure S4E) in all iPSC-derived DA neurons. However, the percentage of β-gal^+^ cells (Figure S4D) and lysosomal accumulation (Figure S4E) increased progressively along with the copy number of *SNCA* both in mock condition and upon SARS-CoV-2 infection. SNCA 4 copy iPSC-derived DA neurons with SARS-CoV-2 infection showed the highest β-gal activity and lysosomal content (Figures S4D and S4E). There is previous evidence that α-Syn has a higher binding affinity to SARS-CoV-2 N protein and that SARS-CoV-2 proteins accelerate the aggregation of α-Syn.^28^^,^^29^ Western blotting confirmed that SARS-CoV-2 infection increases total α-Syn expression and SARS-CoV-2 infected *SNCA* 4 copy iPSC-derived DA neurons showed the highest α-Syn expression (Figure S4F).

### High-throughput screening to identify drug candidates blocking SARS-CoV-2 induced senescence of DA neurons

**Figure 4:**
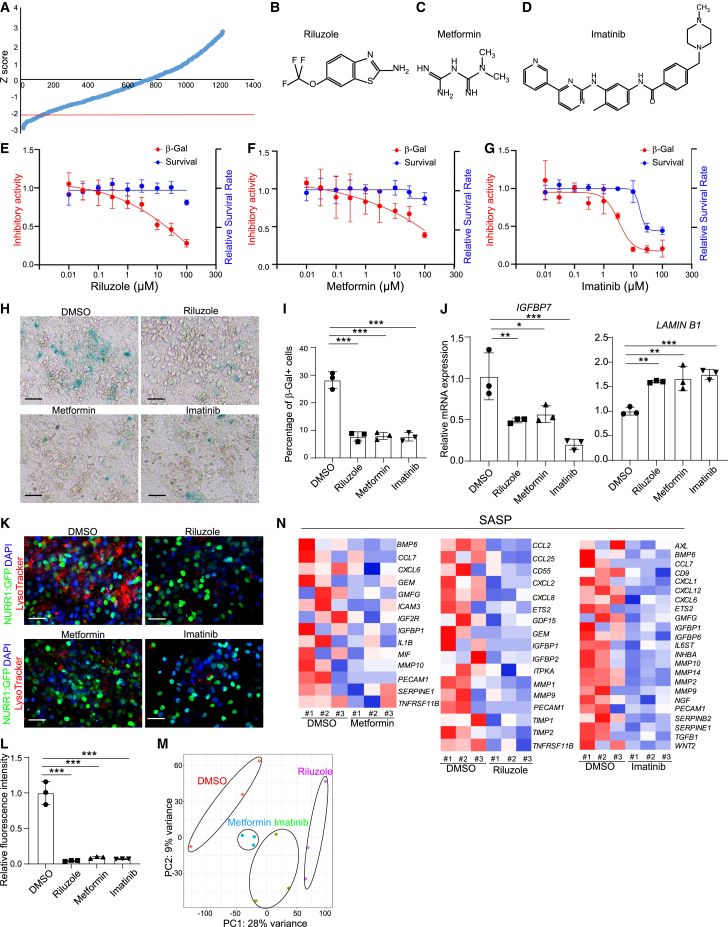
Riluzole, metformin, and imatinib rescue SARS-CoV-2 induced senescence of DA neurons (A) Primary screening results. x axis is the compound number. y axis is the *Z* score. Red line is *Z* score < −2, which means the luminescent signal is lower than average-2 × SD. (B–D) Chemical structures of riluzole (B), metformin (C), and imatinib (D). (E–G) Efficacy and cytotoxicity curves of riluzole (E), metformin (F), and imatinib (G). n = 3 independent biological replicates. (H and I) β-gal staining (H) and quantification of the percentage of β-gal^+^ cells (I) of DMSO or drug candidates-treated purified NURR1:GFP H9-DA neurons at 72 hpi (MOI = 0.1). Scale bars, 100 μm. n = 3 independent biological replicates. (J) Real-time quantitative PCR analysis of senescence related genes of DMSO or drug candidates-treated purified NURR1:GFP H9-DA neurons at 48 hpi (MOI = 0.1). n = 3 independent biological replicates. (K and L) Immunostaining (K) and quantification of LysoTracker intensity (L) of DMSO or drug candidates-treated purified NURR1:GFP H9-DA neurons at 72 hpi (MOI = 0.1). Scale bars, 100 μm. n = 3 independent biological replicates. (M) PCA plot of gene expression profiles of DMSO or drug candidates-treated purified NURR1:GFP H9-DA neurons at 48 hpi (MOI = 0.1). (N) Heatmap of SASP associated genes of DMSO or drug candidates-treated purified NURR1:GFP H9-DA neurons at 48 hpi (MOI = 0.1). Data were presented as mean ± SD. p values were calculated by one-way ANOVA using Dunnett’s test with a setup control. ^∗^p < 0.05, ^∗∗^p < 0.01, and ^∗∗∗^p < 0.001. See also Figure S5.

To identify drug candidates that may protect DA neurons from SARS-CoV-2-induced senescence, we screened DA neurons against a library of Food and Drug Administration (FDA)-approved drugs supplied at 10 μM. 6 h post-treatment, H9 hPSC-derived DA neurons were infected with SARS-CoV-2 at MOI = 0.2 and analyzed at 72 hpi for β-gal activity. The hPSC-derived DA neurons used for drug screening showed a purity of about 80% NURR1-GFP^+^ cells (Figures S5A and S5B). Compounds with a *Z* score < −2 were defined as primary hit drugs (Figure 4A). The hits were further evaluated for potency and cytotoxicity at different concentrations. Three drugs, riluzole (Figures 4B and 4E), metformin (Figures 4C and 4F), and imatinib (EC_50_ = 3.25 μM, CC_50_ = 17.14 μM, Figures 4D and 4G), reduced β-gal activity in a dose-dependent manner without inducing cytotoxicity. Next, we confirmed the drugs’ activities on DA neurons derived from the purified NURR1-GFP^+^ cells. DA neurons treated with either 10 μM riluzole, 50 μM metformin, or 10 μM imatinib showed a significant decrease in the percentage of β-gal^+^ cells as compared with DMSO treatment (Figures 4H and 4I). Real-time quantitative PCR analysis showed a decrease in the senescence-pathway associated gene *IGFBP7* and an upregulation of *LMNB1* for each of the three drugs (Figure 4J). Moreover, these three drugs also decreased lysosomal accumulation compared with DMSO-treated cells (Figures 4K and 4L).

RNA-seq analysis was applied to determine the transcriptional changes induced by the drug candidates versus DMSO in DA neurons upon SARS-CoV-2 infection. Plotting these datasets by PCA (Figure 4M) and by performing clustering analysis (Figures S5C–S5E) demonstrated that DA neurons treated with drug candidates + SARS-CoV-2 occupied a distinct transcriptional space compared with DMSO + SARS-CoV-2-treated DA neurons. Importantly, the genes involved in SASP phenotype (Figure 4N) and senescence pathway (Figures S5F–S5H) were downregulated in riluzole, metformin, or imatinib treated DA neurons in the presence of SARS-CoV-2 infection.

### Riluzole, metformin, and imatinib blocking SARS-CoV-2 infection induced DA neuron senescence by blocking SARS-CoV-2 infection

**Figure 5:**
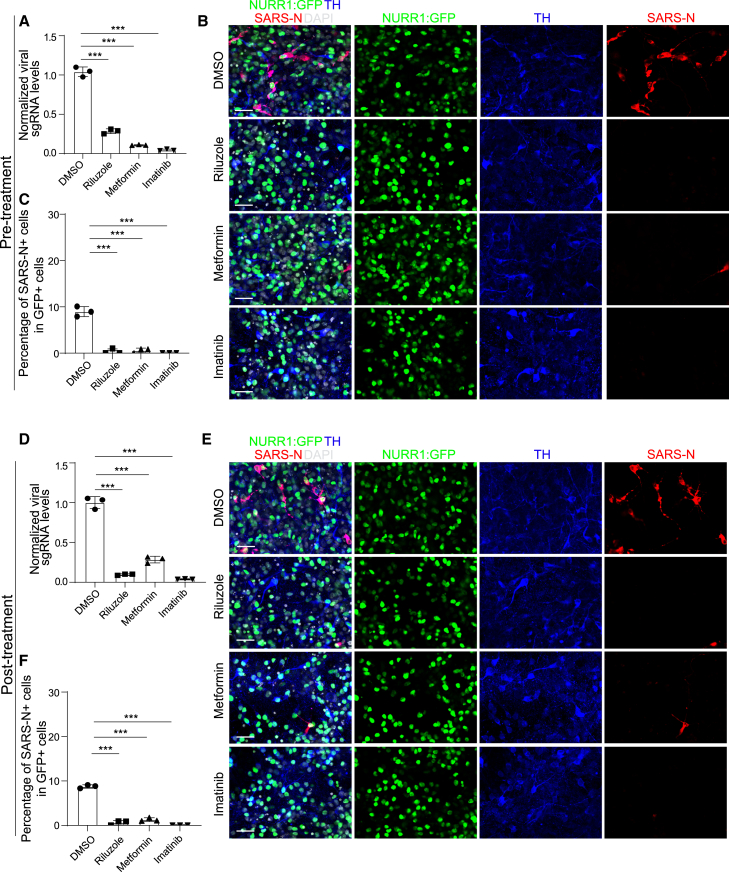
Riluzole, metformin, and imatinib block SARS-CoV-2 infection (A) Real-time quantitative PCR analysis of total RNA from DMSO or drug candidates-pre-treated purified NURR1:GFP H9-DA neurons following SARS-CoV-2 infection (MOI = 0.2) for viral N sgRNA at 48 hpi. The graph depicts the mean sgRNA level normalized to *ACTB*. n = 3 independent biological replicates. (B and C) Representative confocal images (B) and quantification of the percentage of SARS-N^+^ cells (C) of DMSO or drug candidates-pre-treated purified NURR1:GFP H9-DA neurons following SARS-CoV-2 infection (MOI = 0.2) at 72 hpi. Scale bars, 50 μm. n = 3 independent biological replicates. (D) Real-time quantitative PCR analysis of total RNA from DMSO or drug candidates-post-treated purified NURR1:GFP H9-derived DA neurons after SARS-CoV-2 infection (MOI = 0.2) for viral N sgRNA at 48 hpi. The graph depicts the mean sgRNA level normalized to *ACTB*. n = 3 independent biological replicates. (E and F) Representative confocal images (E) and quantification of the percentage of SARS-N^+^ cells (F) of DMSO or drug candidates-post-treated purified NURR1:GFP H9-DA neurons after SARS-CoV-2 infection (MOI = 0.2) at 72 hpi. Scale bars, 50 μm. n = 3 independent biological replicates. Data were presented as mean ± SD. p values were calculated by one-way ANOVA using Dunnett’s test with a setup control. ^∗∗∗^p < 0.001. See also Figure S6.

The lead compounds might decrease senescence by blocking SARS-CoV-2 infection or by rescuing senescence pathway directly. To distinguish between these two possibilities, DA neurons were again treated with 10 μM riluzole, 50 μM metformin, or 10 μM imatinib and then infected with SARS-CoV-2 (pre-treatment). At 48 hpi, real-time quantitative PCR analysis demonstrated that riluzole, metformin, and imatinib all decreased viral RNA (Figure 5A), a finding further validated by immunostaining using an antibody against the SARS-CoV-2 nucleocapsid protein (Figures 5B and 5C). To determine the therapeutic potential of the drug candidates, we infected DA neurons with SARS-CoV-2 first, and then treated cells with 10 μM riluzole, 50 μM metformin, or 10 μM imatinib at 4 hpi (post-treatment). Consistent with pre-treatment, the candidate drugs significantly decreased both replicating viral RNA (Figure 5D) and the percentage of SARS-N^+^ cells (Figures 5E and 5F) when applied at the post-infection time point. Moreover, these three drug candidates also blocked virus infection in MEL-1-derived DA neurons (Figures S6A–S6C). To examine if these drugs can rescue senescence phenotype independent of virus infection, we used SATB1 knockout (KO) hPSCs, which have been reported to show senescence phenotype when differentiated toward DA neurons.^8^ Neither of these three drugs decreased β-gal staining or significantly changed the expression of *IGFBP7* and *LMNB1*, suggesting that these three drugs do not directly rescue the SATB1 KO-induced senescence phenotype (Figures S6D and S6E). Together, this suggests that riluzole, metformin, and imatinib prevent SARS-CoV-2 infection induced DA neuron senescence by blocking SARS-CoV-2 infection.

**Figure 6:**
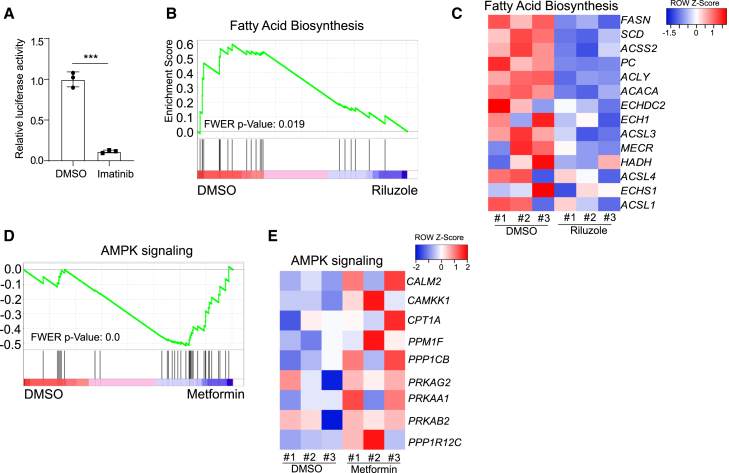
Mechanisms of three drug candidates block SARS-CoV-2 infection (A) Luciferase activity in lysates from DMSO or imatinib treated purified NURR1:GFP H9-derived DA neurons at 24 hpi following exposure to SARS-CoV-2-entry virus at MOI = 0.01. n = 3 independent biological replicates. Data were presented as mean ± SD. p values were calculated by unpaired two-tailed Student’s t test. ^∗^p < 0.05. (B) Gene set enrichment analysis (GSEA) of fatty acid biosynthesis pathway in DMSO or riluzole treated purified NURR1:GFP H9-derived DA neurons at 48 hpi of SARS-CoV-2 (MOI = 0.2). (C) Heatmap of fatty acid biosynthesis pathway associated genes in DMSO or riluzole treated purified NURR1:GFP H9-derived DA neurons at 48 hpi of SARS-CoV-2 (MOI = 0.2). (D) Gene set enrichment analysis (GSEA) of AMPK signaling pathway in DMSO or metformin treated purified NURR1:GFP H9-derived DA neurons at 48 hpi of SARS-CoV-2 (MOI = 0.2). (E) Heatmap of AMPK signaling pathway associated genes in DMSO or metformin treated purified NURR1:GFP H9-derived DA neurons at 48 hpi of SARS-CoV-2 (MOI = 0.2). See also Figure S6.

We further examined the molecular mechanisms of the anti-viral activities of these three drugs. Our previous studies have reported imatinib can block SARS-CoV-2 viral entry in lung organoids through binding ACE2 receptor.^30^ Luciferase activity assay following infection of SARS-CoV-2-entry virus to DA neurons showed decreased luciferase activity in imatinib treated condition compared to mock, indicating that imatinib can block SARS-CoV-2 viral entry in DA neurons (Figure 6A). GSEA identified the downregulation of fatty acid biosynthesis pathway in riluzole-treated condition (Figures 6B and 6C). Several studies have shown the important role of fatty acid biosynthesis in SARS-CoV-2 infection,^29^^,^^31^^,^^32^ suggesting that riluzole might block SARS-CoV-2 infection by inhibiting fatty acid biosynthesis. GSEA also identified the upregulation of the adenosine monophosphate-activated protein kinase (AMPK) pathway upon metformin treatment (Figures 6D and 6E). Consistent with our findings, a recent study identified metformin as a potential COVID-19 therapeutic agent and implicated increased AMPK signaling as protective to SARS-CoV2.^33^ We also tested the three drug candidates on hPSC-derived cortical neurons and did not detect obvious cell cytotoxicity at concentrations effective to protect DA neurons (Figure S6F).

### SARS-CoV-2 is detected in autopsy substantia nigra samples of COVID-19 patients

**Figure 7:**
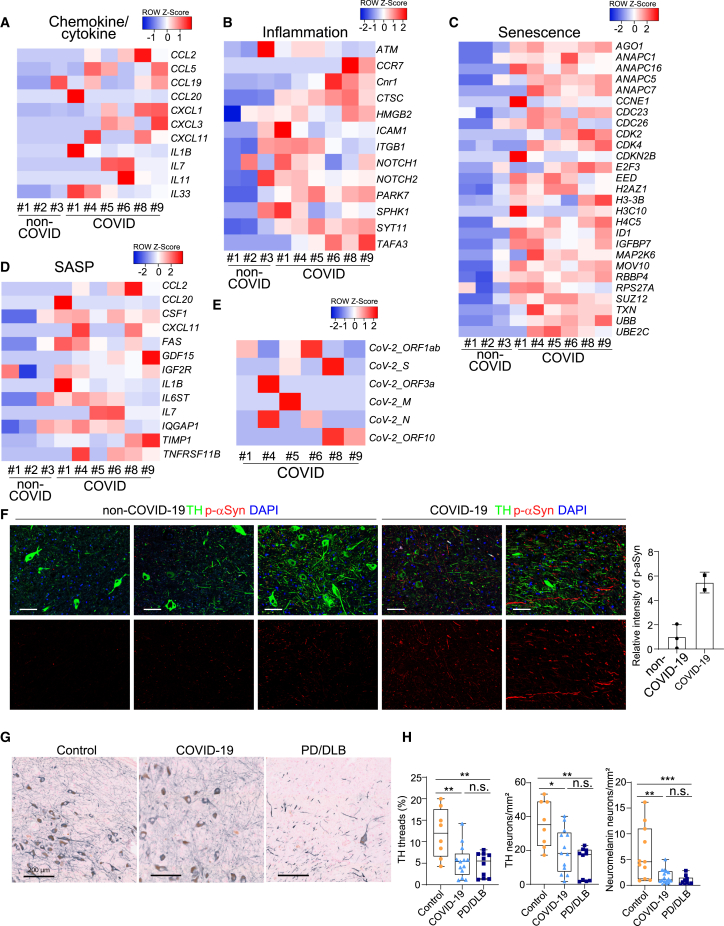
Inflammatory, senescence, and DA neuron degenerative phenotypes were detected in autopsy substantia nigra samples of COVID-19 patients (A–D) Heatmap of chemokine/cytokine (A), inflammation-associated genes (B), senescence-associated genes (C), and SASP associated genes (D) in the autopsy substantia nigra sections of COVID-19 patients versus non-COVID-19 patients (cohort 1: N = 6 COVID-19 patients; N = 3 non-COVID-19 patients). (E) Heatmap of viral transcripts in autopsy substantia nigra sections of COVID-19 patients (cohort 1: N = 6 COVID-19 patients; N = 3 non-COVID-19 patients). (F) Representative confocal images (left) and quantification (right) of p-aSyn in the autopsy substantia nigra sections of COVID-19 patients versus non-COVID-19 patients (cohort 1: N = 2 COVID-19 patients; N = 3 non-COVID-19 patients). Scale bars, 50 μm. (G) Representative images of TH-immunoreactivity (gray) in the substantia nigra (cohort 2). Scale bars, 200 μm. Data were presented as mean ± SD. p values were calculated by unpaired two-tailed Student’s t test. (H) Graphs illustrating quantification of neuromelanin-containing neurons, TH-immunoreactive neurons, and threads (cohort 2). p values were calculated by a Kruskal-Wallis test. ^∗^p < 0.05, ^∗∗^p < 0.01, and ^∗∗∗^p < 0.001. n.s., no significant. See also Figure S7 and Tables S1, S2, S3, and S4.

A key question is whether the selective vulnerability of hPSC-derived DA neurons and the resulting senescence and inflammatory responses are reflected in any cognate changes in the brain of human COVID-19 patients. To answer the question, we first collected human substantia nigra autopsy samples from 6 COVID-19 patients and 3 age-matched controls (cohort 1). Immunostaining for TH was used to confirm substantia nigra identity and the presence of DA neurons (cohort 1, Figure S7A). Next, we performed RNA-seq analysis on RNA isolated from formalin-fixed paraffin-embedded (FFPE) autopsy samples of 6 COVID-19 patients and 3 age-matched controls. Remarkably, the same transcriptional signatures identified in SARS-CoV-2 infected DA neurons *in vitro* (Figures S3I, S3J, S3K, and 3C) were observed in COVID-19 autopsy samples, including the induction of chemokine/cytokine (cohort 1, Figure 7A), inflammation (cohort 1, Figure 7B), senescence-associated (cohort 1, Figure 7C) genes, and SASP associated genes (cohort 1, Figure 7D). The RNA-seq data also showed expression for several SARS-CoV-2 transcripts across the 6 substantia nigra samples from severe COVID-19 patients, compatible with the presence of virus (cohort 1, Figure 7E). Immunostaining showed the co-localization of SARS-N and DA neuron marker TH (cohort 2, Figure S7B). However, we also detected very low levels of viral RNA by real-time quantitative PCR in frozen tissue samples from other brain regions from these same autopsies (data not shown), which could potentially represent virus in leptomeningeal or intracerebral vessels.^34^ Immunostaining showed accumulation of p-a-Syn (cohort 1, Figure 7F) in COVID-19 samples compared with the expression in non-COVID-19 samples, which indicates a potential link of PD phenotypes within the substantia nigra of COVID-19 patients despite the lack of acute clinical symptoms specific to midbrain dysfunction.^34^

We also collected another set of human substantia nigra samples, including 8 controls, 13 COVID-19, and 8 PD(D) donors, to assess whether the DA neurons in the substantia nigra pars compacta are damaged or reduced in number due to SARS-CoV-2 infection (cohort 2). We quantified neuromelanin (NM)-positive neurons, TH-immunoreactive neurons, and TH-immunoreactive threads using an in-house QuPATH script. Demographics, main clinical and pathological features of the 8 controls, 13 COVID-19, and 8 PD(D) donors in cohort 2 are presented in Tables S1, S2, and S3. None of cohort 2 COVID-19 patients had obvious PD symptoms before testing positive with SARS-CoV-2 infection. The percentage of males was different between groups (50%, 84%, and 25% in controls, COVID-19, and PD(D), respectively). Age at death was not significantly different between groups (p > 0.05). Disease duration for COVID-19 was between 5 and 30 days in the COVID-19 groups, whereas the disease duration for PD in the PD(D) was between 10 and 17 years after the start of subjective complaints. SARS-CoV-2 infection was confirmed in the COVID-19 donors using a quantitative PCR test at the time of hospital submission. As by definition, the PD(D) group showed higher Braak aSyn stages, Braak neurofibrillary stages, and CERAD scores and higher levels of AD-related pathologies (all p < 0.001) than COVID-19 and control group. The density of NM-containing and TH-immunoreactive neurons was decreased in COVID-19 and PD(D) brain donors compared with age-matched controls (NM+: −58%, p = 0.008% and −71%, p < 0.001, respectively; TH+: −44%, p = 0.035; −64%, p < 0.002). The percentage area of TH-immunoreactivity threads was also decreased in COVID-19 and PD(D) donors (TH+ −55%, p = 0.006; 60%, p = 0.006, respectively) (cohort 2, Figures 7G and 7H). Although follow-up studies in additional, independent cohorts of severe COVID-19 patients will be needed to further corroborate our results, the overall findings of our study on the selective vulnerability of hPSC-derived DA neurons *in vitro*, the associated inflammatory and cell senescence responses observed in DA neurons *in vitro*, and the inflammatory, cell senescence, and potentially degenerative responses in COVD-19 patient samples *in vivo* argue that these results may be of clinical relevance.

## Discussion

Advancements in hPSC technology allow for the study of host-virus interactions in human, disease-relevant cells.^35^ Previous work using hPSC-derived models has established that choroid plexus cells within the CNS are highly susceptible to SARS-CoV-2 infection,^3^^,^^4^ whereas the tropism of SARS-CoV-2 for neurons has remained unclear.^3^^,^^4^^,^^5^^,^^6^ Here, we differentiated DA neurons from hPSCs using a published protocol.^8^^,^^9^ Using NURR1-GFP^+^ sorted cells at day 25 of the differentiation, we confirmed that SARS-CoV-2 can infect DA neurons. Since the majority of the cells are DA neurons as indicated by immunostaining, single-cell real-time quantitative PCR, and scRNA-seq, we do not expect other cell types to meaningfully contribute to the reported senescence phenotypes. scRNA-seq analysis indicates that SARS-CoV-2 infects DA neurons characterized by high expression of *LMO3* and a lack of *CALB1* expression. Therefore, DA neurons with an A9-like subtype identity may be particularly vulnerable to SARS-CoV-2, similar to the vulnerability of A9 neurons in the substantia nigra most affected in PD.^12^

Here, we report that SARS-CoV-2 infection triggers cellular senescence in DA neurons. Previous work indicates that senescence of DA neurons can function as a contributing factor in PD pathogenesis.^8^ As DA neuron dysfunction is also linked to lethargy and anhedonism,^36^ its role in the post-COVID lethargy/syndrome or long COVID may deserve further study.

Interestingly, *SNCA* 4 copy iPSC-derived DA neurons show increased SARS-CoV-2 infection compared with the isogenic 2 copy or 0 copy iPSC-derived DA neurons. This might be due to the increased TLR4 expression, which has been reported to facilitate viral entry.^26^^,^^27^^,^^37^ Consistently, *SNCA* 4 copy iPSC-derived DA neurons infected by SARS-CoV-2 showed the highest β-gal activity, lysosome accumulation, and total α-Syn, suggesting the potential synergistic or add-on activities of SARS-CoV-2 infection and PD causal mutations.

The FDA-approved drugs, riluzole, metformin, and imatinib, were identified to block SARS-CoV-2-mediated DA neuron senescence, which might function through inhibiting viral infection. Although imatinib was also identified to block SARS-CoV-2 entry in our hPSC-derived lung organoid-based screen,^30^ riluzole has not been previously linked to SARS-CoV-2 infection. The use of metformin has been associated with a decrease in the mortality of COVID-19 patients with obesity and/or type 2 diabetes.^38^^,^^39^ Recently, metformin has been identified as a potential COVID-19 therapeutic agent, and we found it can activate AMPK signaling pathway in DA neurons, which is consistent with recently published papers.^33^ However, we cannot exclude other, yet unknown, mechanisms for the anti-viral activities of these three drugs. Future studies are needed to dissect the mode of action and translational potential of these compounds and whether they are capable of reversing infection-induced neuropathology.

Overall, our data highlight DA neurons as a possible target for SARS-CoV-2 infection, which in turn may trigger an inflammatory and cellular senescence response. Although we observed a comparable inflammatory and senescence signature in SARS-CoV-2 infected hPSC-derived DA neuron cultures *in vitro* and in autopsy samples *in vivo* (cohort 1), we cannot exclude the possibility that other cell types such as astrocytes or microglia or other pathological changes such as hypoxic state could contribute to the inflammatory and senescence signatures in the substantia nigra samples. Given our findings, we posit that over the coming years, there is a need to closely monitor COVID-19 patients for an increased risk of developing PD-related symptoms.

### Limitations of the study

The detection of SARS-CoV-2 viral antigen in brain autopsy samples is highly controversial. Although we are currently still trying to generate EM to assess evidence for direct viral infection in substantia nigra neurons, those studies are technically extremely challenging given the low number of DA neurons and difficulties in sample availability and quality. In addition, due to the biosafety requirements of COVID-19 autopsy samples, we are not capable of performing extensive analysis, such as scRNA-seq, on autopsy samples of COVID-19 patients. The co-staining of the TH neuron marker and SARS-N viral antigen was initially conducted on an autopsy sample, but further validation with a larger sample size is necessary to confirm these findings and to rule out technical challenges such as senescence-related lipofuscin expression that could interfere with reliable SARS-N detection. Furthermore, our study represents the first detailed, quantitative pathological analysis of midbrain DA neurons in severe COVID-19 patients and is limited to the cohort 2 of patients presented here, representing the first wave of COVID-19 cases. Additional analyses using independent and more recent cohorts of patients are needed to further validate the inflammatory and degenerative phenotypes observed. Finally, by focusing on cellular senescence in 72-h infection model, there is the possibility that the observed phenotypes may be self-limiting, arising in response to acute stress, and potentially resolving with cessation of infection.

In this study, we have shown that DA neurons can be infected by SARS-CoV-2 virus. However, only a small percentage of cells (∼5%) are infected at the experimental endpoint (48 hpi). This may be due to bystander effects, as we cannot exclude the possibility that the production of interferons by the initially infected cells protects surrounding cells against subsequent rounds of infections. Nevertheless, infected DA neurons exhibit a senescence phenotype that results in the secretion of toxic cytokines, which may trigger a SASP. SASP may further cause inflammation and disrupt tissue structure and function locally and contribute to chronic inflammation throughout the body. However, the clinical relevance the level of individual patients is as of yet unknown given that the infection appears to affect only a relatively small percentage of DA neurons.

## STAR★Methods

### Key resources table

**Table:** 

REAGENT or RESOURCE	SOURCE	IDENTIFIER
**Antibodies**		

Anti-SARS-CoV/SARS-CoV-2 Nucleocapsid	Sino Biological	Cat#40143-R001; RRID:AB_2827974
Anti-ACE2	Abcam	Cat #ab15348; RRID:AB_301861
Firefly luciferase Monoclonal Antibody (CS 17)	Thermo Fisher Scientific	Cat #35-6700; RRID: AB_2533218
Anti-Alpha Synuclein	Millipore	Cat #S5566-100UL; RRID:AB_261518
Anti-MAP2 antibody	Abcam	Cat #ab5392; RRID:AB_2138153
p21 Waf1/Cip1 (12D1) Rabbit mAb	Cell Signaling	Cat #2947; RRID:AB_823586
Anti-α-Synuclein Phospho	Biolegend	Cat #825701; RRID:AB_2564891
Goat polyclonal anti-FOXA2	R&D Systems	Cat # AF2400; RRID:AB_2294104
Recombinant Anti-TBR1 antibody	Abcam	Cat # ab183032; RRID:AB_2936859
Anti-Ctip2 antibody	Abcam	Cat #ab18465; RRID:AB_2064130
Anti-Tyrosine Hydroxylase antibody - Neuronal Marker	Abcam	Cat #ab112; RRID:AB_297840
Human/Mouse Tyrosine Hydroxylase Antibody	R&D Systems	Cat #MAB7566; RRID:AB_2923064
Anti-FOXA2 Antibody	Santa Cruz	Cat #sc-6554; RRID:AB_2262810
Anti-GFP antibody	Abcam	Cat #ab13970; RRID:AB_300798
Human Lamin B1 Antibody	R&D Systems	Cat #MAB8525; RRID:AB_3075539
Rabbit polyclonal anti-LMX-1	Millipore	Cat #AB10533; RRID:AB_10805970
Donkey anti-Mouse IgG (H+L) Cross-Adsorbed Secondary Antibody, Alexa Fluor 488	Thermo Fisher Scientific	Cat #A-21202; RRID:AB_141607
Donkey anti-Mouse IgG (H+L) Secondary Antibody, Alexa Fluor 594 conjugate	Thermo Fisher Scientific	Cat #A-21203; RRID:AB_141633
Donkey anti-Mouse IgG (H+L) Secondary Antibody, Alexa Fluor 647	Thermo Fisher Scientific	Cat #A-31571; RRID:AB_162542
Alexa Fluor 488 AffiniPure Donkey Anti-Chicken IgY (IgG) (H+L)	Jackson Immunoresearch Labs	Cat #703-545-155; RRID:AB_2340375
HRP Western Blot Anti-rabbit IgG Antibody	Thomas Scientific	Cat #KCB003; RRID:AB_10702763
HRP Western Blot Anti-Mouse IgG Antibody	Thomas Scientific	Cat #KCB002; RRID:AB_10703407
Donkey anti-Rabbit IgG (H+L) Secondary Antibody, Alexa Fluor 594	Thermo Fisher Scientific	Cat #A-21207; RRID:AB_141637
Donkey anti-Goat IgG Secondary Antibody, Alexa Fluor 555	Thermo Fisher Scientific	Cat #A32816; RRID:AB_2762839
Donkey anti-Goat IgG (H+L) Cross-Adsorbed Secondary Antibody, Alexa Fluor 647	Thermo Fisher Scientific	Cat #A-21447; RRID:AB_2535864
Donkey anti-Rabbit IgG Secondary Antibody, Alexa Fluor 647	Thermo Fisher Scientific	Cat #A32795; RRID:AB_2762835

**Bacterial and virus strains**		

SARS-CoV-2: hCoV/USA/WA1/2020	WRCEVA (UTMB Health)	https://www.utmb.edu/wrceva/viruses
SARS-CoV-2-entry Viruses	This study	This study

**Biological samples**		

Human samples and refer to Table S4 for all further details		N/A

**Chemicals, peptides, and recombinant proteins**		

Riluzole	Sigma Aldrich	#R116
Imatinib	Sigma Aldrich	#CDS022173
Metformin hydrochloride	Sigma Aldrich	#PHR1084
Vitronectin (VTN-N)	Thermo Fisher Scientific	#A14700
Accutase	Innovative Cell Technologies	#AT104-500
0.5M EDTA, pH 8.0	Thermo Fisher Scientific	#15575-020
L-Glutamine (100X)	Thermo Fisher Scientific	#25030-081
Penicillin Streptomycin	Thermo Fisher Scientific	#15140-122
DMEM F12 (1:1) Ham	Thermo Fisher Scientific	#11320-033
Essential 6 (E6)	Thermo Fisher Scientific	#A1516401
Essential 8 (E8)	Thermo Fisher Scientific	#A1517001
Neurobasal	Life Technologies	#21103-049
N2 supplement B	Stem Cell Technologies	#7156
B27	Life Technologies	#12587-010
XAV-939 (XAV)	Tocris	#3748
Y-27632 (ROCKi)	R&D	#1254
SB431542 (SB)	R&D	#1614
LDN193189 (LDN)	Stemgent	#04-0074
CHIR99021	R&D	#4432
SHH C25II	R&D	#464-SH
brain-derived neurotrophic factor (BNDF)	R&D	#248-BD
ascorbic acid (AA)	Sigma	#4034
dibutyryl cAMP (cAMP)	Sigma	#4043
glial cell line-derived neurotrophic factor (GDNF)	Peptrotech	#450-10
DAPT	R&D	#2634
transforming growth factor type β3 (TGFβ3)	R&D	#243-B3
Poly-L-Ornithine (PO)	Sigma Aldrich	#P3655
Mouse Laminin I (LAM)	R&D	#3400-010-1
Fibronectin (FN)	Thermo Fisher Scientific	#356008
Geltrex	Life Technologies	#A1413201
STEM-CELLBANKER™	Amsbio	#11890
Trizol	Thermo Fisher Scientific	#15596026

**Critical commercial assays**		

LysoTracker™ Red DND-99	Thermo Fisher Scientific	#L7528
MitoTracker™ Deep Red FM	Thermo Fisher Scientific	# M22426
CellROX™ Deep Red Reagent	Thermo Fisher Scientific	# C10422
Senescence β-Galactosidase Staining Kit	Cell Signaling	# 9860S

**Deposited data**		

RNA-seq	This study	GSE174745
scRNA-seq	This study	GSE248989

**Experimental models: Cell lines**		

293T	ATCC	#CRL-11268
Vero E6	ATCC	# CRL-1586 RRID: CVCL_0574
Vero-hACE2-TMPRSS2	BEI Resources	#NR-54970
MEL-1 hESC line	Stem Cells Ltd	NIHhESC-11-0139
H9 (WA-09) hESC line	WiCell Research Institute	NIHhESC-10-0062
NURR1:GFP H9	MSKCC	Riessland et al.^8^
SNCA 0 copy	University of Edinburgh	Chen et al.^17^
SNCA 2 copy	University of Edinburgh	Chen et al.^17^
SNCA 4 copy	University of Edinburgh	Chen et al.^17^

**Oligonucleotides**		

Primers for real-time quantitative PCR and refer to Table S6 for all further details		N/A

**Software and algorithms**		

Rstudio	Rstudio	https://rstudio.com
Seurat R package v3.1.4		https://satijalab.org/seurat/
Dplyr package	LHRI	https://dplyr.tidyverse.org/
Adobe illustrator CC2017	Adobe	https://www.adobe.com/product/photoshop.html
Graphpad Prism 6	Graphpad software	https://www.graphpad.com
Deposited custom code	This study	https://doi.org/10.5281/zenodo.10373669

### Resource availability

#### Lead contact

Further information and requests for resources, reagents or codes should be directed to and will be fulfilled by the lead contact, Shuibing Chen (shc2034@med.cornell.edu).

#### Materials availability

This study did not generate new unique reagents.

#### Data and code availability

Single-cell RNA seq data and RNA-seq data have been deposited at GEO and are publicly available as of the date of publication. Accession numbers are listed in the key resources table. All original code has been deposited at Github and is publicly available as of the date of publication. DOI is listed in the key resources table. Any additional information required to reanalyze the data reported in this paper is available from the lead contact upon request.

### Experimental model and study participant details

#### Human subjects

A total of 31 clinically defined and pathologically confirmed brain donors were included in Cohort 2: 8 PD, 13 COVID-19, and 8 age-matched control donors. During life, all donors provided written informed consent for the use of their brain tissue and medical records for research purposes. PD donors were included in collaboration with the Netherlands Brain Bank (NBB; http://brainbank.nl). Demographic features and clinical symptoms were abstracted from the clinical files, including sex, age-at-onset, age at death, disease duration, presence of dementia. Based on available clinical information, PDD was diagnosed if dementia developed at least a year after the onset of the motor symptoms. The controls were included at the department of Anatomy and Neurosciences, Amsterdam UMC, following the Normal Aging Brain Collection Amsterdam (NABCA; http://nabca.eu) pipeline. All donors underwent brain autopsy and dissection and neuropathological diagnosis by an expert neuropathologist according to the international guidelines of the Brain Net Europe (BNE) consortium. Based on the BNE sampling protocol, the right SN block was cut at the level of the midbrain. The block was subsequently paraffin embedded, followed by immunohistochemistry. Braak and McKeith αSyn stages were determined using the BrainNet Europe (BNE) criteria. Based on Thal amyloid-β phases scored on the medial temporal lobe, Braak neurofibrillary stages and Consortium to Establish a Registry for Alzheimer's Disease (CERAD) neuritic plaque scores, levels of AD pathology were determined according to on NIA-AA consensus criteria. See Tables S1, S2, and S3 for age, gender and more information.

Some brain samples for RNA-seq and immunostaining were taken from a prospective autopsy cohort study, conducted at the Columbia University Presbyterian Hospital and approved by its institutional review board. Informed consent for complete autopsy (including the brain, for which separate and explicit consent was asked) was obtained. In addition, additional samples came from a prospective autopsy cohort study, conducted at Amsterdam University Medical Center, the Netherlands (two locations) and approved by its institutional review board. For both sites, informed consent for complete autopsy (including the brain, for which separate and explicit consent was asked) was obtained. The brain samples were fixed in 4% formaldehyde and routinely processed for paraffin-embedding. Experiments using samples from human subjects were conducted in accordance with local regulations and with the approval of the institutional review board at the Weill Cornell Medicine under protocol METC 2020.167. See Table S4 for age and gender information.

#### Virus strains

##### SARS-CoV-2-entry Viruses

Recombinant Indiana VSV (rVSV) expressing SARS-CoV-2 spikes were generated as previously described.^19^ HEK293T cells were grown to 80% confluency before transfection with pCMV3-SARS-CoV-2-spike (kindly provided by Dr. Peihui Wang, Shandong University, China) using FuGENE 6 (Promega). Cells were cultured overnight at 37°C with 5% CO_2_. The next day, medium was removed and VSV-G pseudo-typed ΔG-luciferase (G^∗^ΔG-luciferase, Kerafast) was used to infect the cells in DMEM at a MOI of 3 for 1 hour before washing the cells with 1×DPBS three times. DMEM supplemented with anti-VSV-G antibody (I1, mouse hybridoma supernatant from CRL-2700; ATCC) was added to the infected cells and they were cultured overnight as described previously.^40^ The next day, the supernatant was harvested and clarified by centrifugation at 300 g for 10 minutes and aliquots stored at −80°C.

##### SARS-CoV-2 Virus

SARS-CoV-2, isolate USA-WA1/2020 was obtained from World Reference Center for Emerging Viruses and Arboviruses located at University of Texas Medical Branch via the CDC. SARS-CoV-2 was propagated in Vero E6 cells (ATCC) in EMEM supplemented with 10% FCS, 1 mM Sodium Pyruvate and 10 mM HEPES as described previously.^40^ All work involving live SARS-CoV-2 was performed in the CDC/USDA-approved BSL-3 facility at Aaron Diamond AIDS Research Center located at Columbia University.

#### Cell lines

Human pluripotent stem cells (hPSCs; WA09 [H9; 46XX] and MEL1 [46XY]], were grown onto Vitronectin (VTN-N, Thermo Fisher #A14700) coated dishes with Essential 8 media (Life Technologies #A1517001). hPSCs were passaged every 4-5 days by EDTA, and passage 35-55 hPSCs were used for the experiments. Generation of NURR1: GFP hESC line was previously described.^8^ Briefly, stop codon of endogenous NR4A2 (NURR1) was replaced by EGFP expression cassette (P2A-H2B-EGFP-PgkPuro) by using a CRISPR/CAS9-mediated knock-in approach. The resulting *NURR1:GFP*^+^ cells almost express TH (a mature mDA marker; 98%) based on single cell real-time quantitative PCR.^8^ PD SNCA- iPSC lines (originally named AST23, AST23-2KO, AST23-4KO) were kindly provided by Dr Tilo Kunath from The University of Edinburgh, and method for CRISPR-based gene knock-out was described in the published paper.^17^

“*Cercopithecus aethiops* Kidney Epithelial Cells Expressing Transmembrane Protease Serine 2, and Human Angiotensin-Converting Enzyme 2 (Vero-TMPRSS2) were obtained from BEI Resources (NR-54970) and cultured in Dulbecco’s Modified Eagle Medium (DMEM) supplemented with 10% fetal bovine serum (FBS) and 100 U/mL penicillin and 100 mg/mL streptomycin and 5 microgram/mL Puromycin.”

Vero E6 (Female, African green monkey [*Cercopithecus aethiops*] kidney) were obtained from ATCC (CRL-1587) and cultured in Dulbecco’s Modified Eagle Medium (DMEM) supplemented with 10% fetal bovine serum (FBS) and 100 U/mL penicillin and 100 mg/mL streptomycin.”

HEK 293T cells (Female) were grown in DMEM media supplemented with 10% FBS, 1% PenStrep (Gibco, Grand Island, NY). All cells were cultured at 37°C/5% CO_2_.

### Method details

#### hPSC differentiation toward DA neurons

Midbrain dopaminergic neuron differentiation were performed using H9, MEL1 and *SNCA* different copy numbers hPSCs, which include NURR1: GFP hPSC. hPSCs were grown on VTN-N (Thermo Fisher Scientific)-coated 6-well plates in E8-essential medium. Cells were maintained at 37°C, 5% CO_2_. hPSCs were differentiated with an optimized protocol from a previously reported study.^8^^,^^9^^,^^41^ To purify DA neurons, DA neuron differentiated cells at day 25 from NURR1:GFP hPSC were sorted by GFP using a BD FACS Aria6 cell sorter in Flow Cytometry Core Facility of MSKCC. The GFP positive sorted cells were further *in vitro* cultured for subsequent experiments until use.

#### SARS-CoV-2-entry Viruses infection

hPSC-derived DA neurons were seeded in 24-well plates, SARS-CoV-2-entry virus was added at the indicated MOIs for 1 hour. Then, the cells were cultured at 37°C with 5% CO_2_. At 24 hpi, cells were fixed for immunohistochemistry or harvested for luciferase assay following the Luciferase Assay System protocol (E1501, Promega).

#### SARS-CoV-2 Virus infections

SARS-CoV-2 infections of hPSC-derived DA neurons were performed in the culture media at the indicated MOIs at 37°C. At the indicated hpi, cells were washed three times with PBS. For RNA analysis cells were lysed in TRIzol (Invitrogen). For immunofluorescence staining cells were fixed in 4% formaldehyde for 60 min at room temperature.

#### Plaque assay

SARS-CoV-2 infections of hPSC-derived DA neurons were performed as before. At 6h, supernatant was removed and the cells were washed with PBS (3X) to remove unbound virus. Cells were overlaid with fresh media and incubated at 37°C/5% CO_2_. Supernatant was collected every 24h post incubation and frozen for infectivity measurement up to 72h.

For infectivity measurement, Vero-ACE2-TMPRSS2 cells were seeded at 20,000 cells/well and incubated at 37°C/5% CO_2_ overnight. Supernatants collected at indicated time points were thawed from -80°C. Serial dilutions of the supernatant were performed from 1:2 dilution to 1:2048 dilution. 100ul of dilutions were overlaid on the overnight monolayer of Vero-ACE2-TMPRSS2 cells. Cells were incubated with virus at 37°C/5%CO_2_ for 70h. Endpoint titers were calculated for each sample by visualizing virus cytopathic effects using light microscopy (ECHO Revolve: 10x objective). Vero-ACE2-TMPRSS2 infected with MOI 0.1 of WA1 virus isolates were used as positive controls for the assay.

#### β-Gal Staining

The identification of senescent cells is based on an increased level of β-galactosidase activity. The assay followed Senescence β-Galactosidase Staining Kit (#9860, CST).

#### Western blotting

Cells were collected in Pierce RIPA buffer (Thermo Fisher Scientific) plus HALT protease inhibitor cocktail (1:100) (Thermo Fisher Scientific) and lysates loaded on 12% NuPage Bis-Tris pre-cast gels (Thermo Fisher Scientific). After separation by electrophoresis, proteins were transferred to 0.2 mm nitrocellulose membranes (Thermo Fisher Scientific). Membranes were blocked with 5% milk in TBS +0.1% Tween and incubated with primary antibody overnight. Information for primary antibodies is provided in Table S5. Membranes were washed and incubated with secondary antibody for 1 h at room temperature in 5% milk-TBS-0.1% Tween and developed using Super-Signal West Pico PLUS chemiluminescent substrate (Thermo Fisher Scientific). Human β-Actin was employed as an internal reference.

#### Immunohistochemistry

In brief, paraffin-embedded tissue blocks of the SN were cut into 6 μm-thick consecutive sections with a microtome and incubated with mouse-anti tyrosine hydroxylase (TH, dilution 1:800 in TBS-Trition 0.1%, Immunostar, Hudson, USA) at 4°C overnight. TH immunoreactivity was visualized with Vector SG grey (Vector, California, United State) followed by counter-staining with fast red (Vector, California, United State), and dehydrated in a series of ethanol, xylene, then mounted with Entellan.

#### Tissue delineation and image analysis

Images were taken using a whole-slide scanner (Vectra Polaris, 20x objective) and quantified using QuPath 0.2.3 (https://qupath.readthedocs.io/en/stable/index.html). The SN was delineated according to the anatomical landmarks on TH-stained sections which were counterstained with fast-red. Briefly, the SN was outlined based on the coordinates from the Atlas of Human Brainstem and previous literature (Paxinos and Huang, 1995^42^; Parkkinen et al., 2011^43^; Dijkstra et al., 2014^44^). In brief, the dorsal border of SN was delineated ventrally to the red nucleus and along the white matter tracts with extension to the dorsal-lateral part of the cerebral peduncle and medially reaching the ventral tegmental area (VTA). The ventral border of SN was depicted by the cerebral peduncle, connecting both the lateral and medial end of the dorsal border.

To analyze the TH immunoreactivity, we used an in-house QuPath script. Briefly, an object classifier was used to identify and count TH positive cells and neuromelanin only cells (without TH staining). Once counted, they were subtracted from the annotation, and then a pixel classifier was used to quantify the % area occupied by TH threads. The following outcome measures were defined for the SN: (1) TH positive cell count, (2) neuromelanin only cell count and (3) TH threads % area.

#### Real-time quantitative PCR

Total RNA samples were prepared from cells and DNase I treated using TRIzol according to the manufacturer’s instructions. To quantify viral replication, measured by the expression of sgRNA transcription of the viral N gene, one-step real-time quantitative PCR was performed using SuperScript III Platinum SYBR Green One-Step qRT-PCR Kit (Invitrogen) with primers specific for the TRS-L and TRS-B sites for the N gene as well as ACTB as an internal reference. Real-time quantitative PCR reactions were performed on an Applied Biosystems QuantStudio 6 Flex Real-Time PCR Instrument (ABI). Delta-delta-cycle threshold (ΔΔCT) was determined relative to ACTB levels and normalized to mock infected samples. Error bars indicate the standard deviation of the mean from three biological replicates. The sequences of primers/probes are provided in Table S6.

#### RNA-Seq

Cell infections were performed at the described MOI in DMEM supplemented with 0.3% BSA, 4.5 g/L D-glucose, 4 mM L-glutamine and 1 μg/ml TPCKtrypsin and harvested 24 hpi. Total RNA was extracted in TRIzol (Invitrogen) according to the manufacturer’s instructions. RNAseq libraries of polyadenylated RNA were prepared using the TruSeq Stranded mRNA Library Prep Kit (Illumina) according to the manufacturer’s instructions and sequenced on an Illumina NextSeq 500 platform. The resulting single end reads were checked for quality (FastQC v0.11.5) and processed using the Digital Expression Explorer 2 (DEE2)^45^ workflow. Adapter trimming was performed with Skewer (v0.2.2).^46^ Further quality control done with Minion, part of the Kraken package.^47^ The resultant filtered reads were mapped to human reference genome GRCh38 using STAR aligner^48^ and gene-wise expression counts generated using the “-quantMode GeneCounts” parameter. BigWig files were generated using the bamCoverage function in deepTools2 (v.3.3.0).^49^

For RNA preparation with human exome enrichment, total RNA samples were prepared from formalin-fixed and paraffin-embedded autopsy substantia nigra tissues followed by DNaseI treatment using manufacturer’s instructions (Qiagen RNeasy FFPE kit Cat# 73604). 100 ng total RNA was prepared using NEB Next Ultra II RNA Library Prep Kit without polyA selection or RNA depletion, then the libraries were enriched with twist human exome probes and reagents.

For RNA preparation with COVID-19 panel enrichment, 100ng total RNA was prepared using NEB Next Ultra II RNA Library Prep Kit without polyA selection or RNA depletion, then the libraries were enriched with IDT COVID-19 Capture Panel probes and reagents.

For analysis, the human genome GRCh38 was used to generate the hisat2 index and then alignment from the raw fastq file. The quantification of reads for each individual gene were counted using feartureCounts. Then the clustering and differential gene expression analysis were using the edgeR package after import the expression and phenotype data in R.

#### Single cell RNA-Seq

The mock and SARS-CoV-2 infected hPSC-derived DA neuron cells were dissociated into single cells using Accutase at 37°C for about 30 min, and then neutralized with culture medium. The dissociated cells were pelleted and resuspended in PBS with 0.04% BSA. The resuspended cells were then placed through a 40 μm filter to obtain a single cell suspension. The single cell suspension was processed with the Chromium Single Cell 5’ Reagent Kit v3 (10x Genomics, # 1000263) using a 10X Genomics Chromium Controller following the instruction of the user guide. The final libraries were assessed by Agilent Technology 2100 Bioanalyzer and sequenced on Illumina NovaSeq sequencer with pair-end 2x100 cycle kit (28+10+10+90).

Sequencing and gene expression UMI count matrix generation The FASTQ files were imported to a 10X - data analysis pipeline (v3.0.2) to align reads, generate feature-barcode matrices and perform clustering and gene expression analysis. In a first step, cellranger mkfastq demultiplexed samples and generated fastq files; in the second step, cellranger count aligned fastq files to the reference genome and extracted gene expression UMI counts matrix. The expression matrix was then imported into Seurat for further clustering analysis. To exclude poor quality cells that might result from cell death or other membrane damages, we filtered cells based on the number of expressed genes detected, the sum of UMI counts and the proportion of mitochondrial genes. We visualized QC metrics and used these to filter cells. We filtered cells that have unique feature counts over 6000 or less than 200 and the cells that have >5% mitochondrial counts. Finally, 4702 cells which passed the quality control were used for the following analysis. We normalized the sum of UMI counts for each cell to the median of all cells. After normalization, the UMI count data were log-transformed. Then, the data were centered for each gene by subtracting the average expression of that gene across all cells. The top enriched genes in each cluster were used to identity the cell types. And the expression levels of selected genes were visualized and demonstrated with the function Vlnplot, FeaturePlot and Dotplot.

#### In situ hybridization

Adherent cells plated in a glass-bottom plate are fixed and permeabilized and stained for a protein of interest (TH; tyrosine hydroxylase) in order to locate RNA puncta signals within a mature DA neuron. Following protein detection, a fluorescent *in situ* hybridization (FISH) and branched DNA amplification technology is used to amplify the signal detection of an RNA transcript. In the first step, a gene-specific oligonucleotide target probe binds to the target RNA sequence. Signal amplification is then achieved through a series of sequential hybridization steps. After two sequential amplifying steps, a fluorescent dye is introduced to hybridize to their corresponding amplifier molecules. RNA signals in dots are visualized using confocal microscopy with 63X oil lenses. All the images in z-stacks were projected and obtained using Imaris software. Projected images were analyzed for quantification.

#### High Throughput Chemical Screening

hPSC-derived DA neurons were cultured in 384-well plates at 10,000 cells/50 μl medium/well until Day 40. Compounds from an in-house FDA-approved drug library (Prestwick) were added at 10 μM. DMSO treatment was used as a negative control. hPSC-derived DA neurons were further infected with SARS-CoV-2 (MOI=0.1). After 72 hpi, hPSC-derived DA neurons were harvested for β-galactosidase assay using Senescence β-Galactosidase Staining Kit (#9860, CST) protocol.

To calculate EC50 and CC50, cells were stained with β-Galactosidase Staining Kit and normalized to DMSO-treated condition. To calculate CC50, the cell survival was monitored by DAPI and normalized to DMSO-treated condition. The efficacy and cytotoxicity curves were calculated using Prism GraphPad Prism 7.0.

### Quantification and Statistical analysis

N=3 independent biological replicates were used for all experiments unless otherwise indicated. Data was presented as mean ± STDEV. For the comparison between two samples, *P* values were calculated by unpaired two-tailed Student’s t test unless otherwise indicated. For the comparison between DMSO or drug candidates-treated samples, *P* values were calculated by one-way ANOVA using Dunnett’s test with a set up control. To examine between-group differences regarding clinical and pathological features, we used independent sample *t*-tests for continuous variables, chi-square tests for categorical variables and Kruskall-Wallis tests for ordinal variables. Group differences in the neuromelanin neuron density, TH density and %TH threads were tested using a KrusKall-Wallis test. Data analysis was performed using GraphPrism version 9.5.1. n.s. indicates a non-significant difference. ^∗^*p*<0.05, ^∗∗^*p*<0.01 and ^∗∗∗^*p*<0.001.

## Supplemental information

Document S1. Figures S1–S7, Tables S4–S6, and supplemental references

Table S1. Demographics and pathological characterization of control autopsy cohort, related to Figures 7 and S7

Table S2. Demographics and pathological characterization of COVID-19 autopsy cohort, related to Figures 7 and S7

Table S3. Demographics and pathological characterization of PD(D) autopsy cohort, related to Figures 7 and S7

Document S2. Article plus supplemental information
