# Supplementary material for: SARS-CoV-2 infection causes dopaminergic neuron senescence
Source: Cell Stem Cell. 2024 Feb 1;31(2):196–211.e6. doi: 10.1016/j.stem.2023.12.012 (PMC10843182; doi:10.1016/j.stem.2023.12.012)
Supplement: Document S1. Figures S1–S7, Tables S4–S6, and supplemental references [file mmc1.pdf]

Cell Stem Cell, Volume *31*

Supplemental Information

SARS-CoV-2 infection causes dopaminergic neuron senescence

Liuliu Yang, Tae Wan Kim, Yuling Han, Manoj S. Nair, Oliver Harschnitz, Jiajun Zhu, Pengfei Wang, So Yeon Koo, Lauretta A. Lacko, Vasuretha Chandar, Yaron Bram, Tuo Zhang, Wei Zhang, Feng He, Chendong Pan, Junjie Wu, Yaoxing Huang, Todd Evans, Paul van der Valk, Maarten J. Titulaer, Jochem K.H. Spoor, Robert L. Furler O'Brien, Marianna Bugiani, Wilma D.J. Van de Berg, Robert E. Schwartz, David D. Ho, Lorenz Studer, and Shuibing Chen

Figure S1

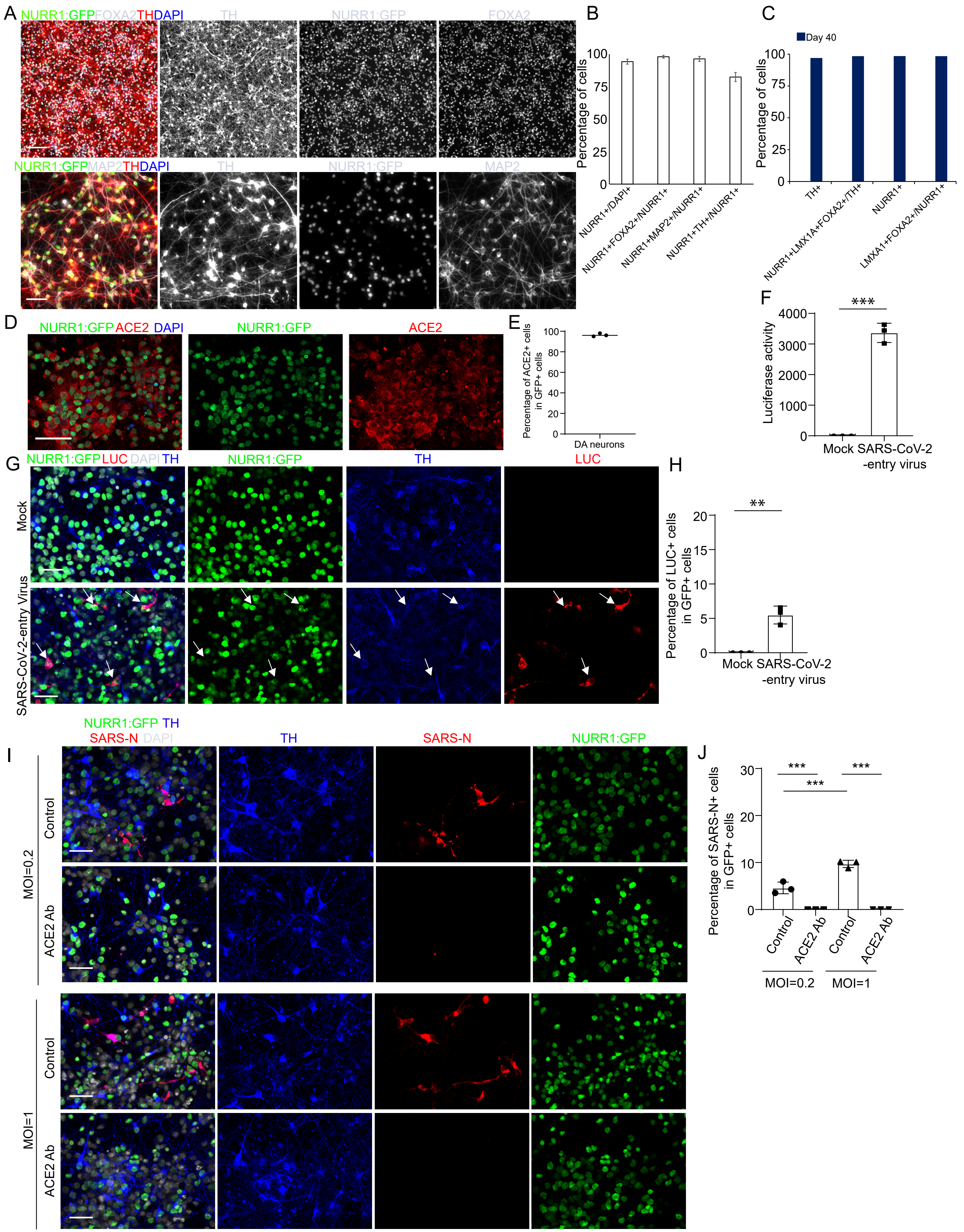
A
NURR1:GFP FOXA2 TH DAPI
TH
NURR1:GFP
FOXA2
NURR1:GFP MAP2 TH DAPI
MAP2
B
Percentage of cells
NURR1+/DAPI+
NURR1+FOXA2+/NURR1+
NURR1+MAP2+/NURR1+
NURR1+TH+/NURR1+
C
Day 40
TH+
NURR1+LMX1A+FOXA2+/TH+
NURR1+
LMXA1+FOXA2+/NURR1+
D
NURR1:GFP ACE2 DAPI
ACE2
E
Percentage of ACE2+ cells in GFP+ cells
DA neurons
F
Luciferase activity
Mock
SARS-CoV-2 -entry virus
G
NURR1:GFP LUC DAPI TH
LUC
Mock
SARS-CoV-2-entry Virus
H
Percentage of LUC+ cells in GFP+ cells
I
NURR1:GFP TH SARS-N DAPI
SARS-N
MOI=0.2
MOI=1
Control
ACE2 Ab
J
Percentage of SARS-N+ cells in GFP+ cells

SUPPLEMENTAL INFORMATION

Figure S1. Characterization of hPSC-derived DA neurons. Related to Figure 1.

(A and B) Representative confocal images (A) and quantification (B) of NURR1: GFP H9-derived DA neurons stained with antibodies recognizing NURR1-GFP, TH, MAP2 or FOXA2. Scale bar=100 μm (upper panel) and 50μm (lower panel). n=3 independently biological replicates.

(C) Single cell RT-qPCR analysis of DA neuron marker genes from FACS based NURR1-GFP^{+} purified cells at day40 (67 cells) from previous study [1].

(D and E) Representative confocal images (D) and quantification of ACE2^{+} cells in NURR1-GFP^{+} cells (E) of purified NURR1: GFP-derived DA neurons stained with ACE2 antibody. Scale bar=50 μm. n=3 independent biological replicates.

(F) Luciferase activity in lysates from purified NURR1: GFP-derived DA neurons at 24 hpi following exposure to SARS-CoV-2-entry virus at MOI=0.01. The Y-axis indicates the absolute luciferase activity between mock and infect groups. n=3 independent biological replicates.

(G and H) Representative confocal images (G) and quantification (H) of purified NURR1: GFP-derived DA neurons infected with SARS-CoV-2-entry virus (MOI=0.01) at 24 hpi using antibodies against luciferase and DA neuron markers. Scale bar=50 μm. n=3 independent biological replicates.

(I and J) Representative confocal images (I) and quantification (J) of purified NURR1: GFP-derived DA neurons infected with SARS-CoV-2 virus at different MOI (MOI=0.2 or MOI=1) and treated with ACE2 blocking antibody at 72 hpi using antibodies against SARS-N and DA neuron markers. Scale bar=50 μm. n=3 independent biological replicates.

Data was presented as mean ± STDEV. *P* values were calculated by unpaired two-tailed Student's t test. $^{**}P < 0.01$, $^{***}P < 0.001$.

Figure S2

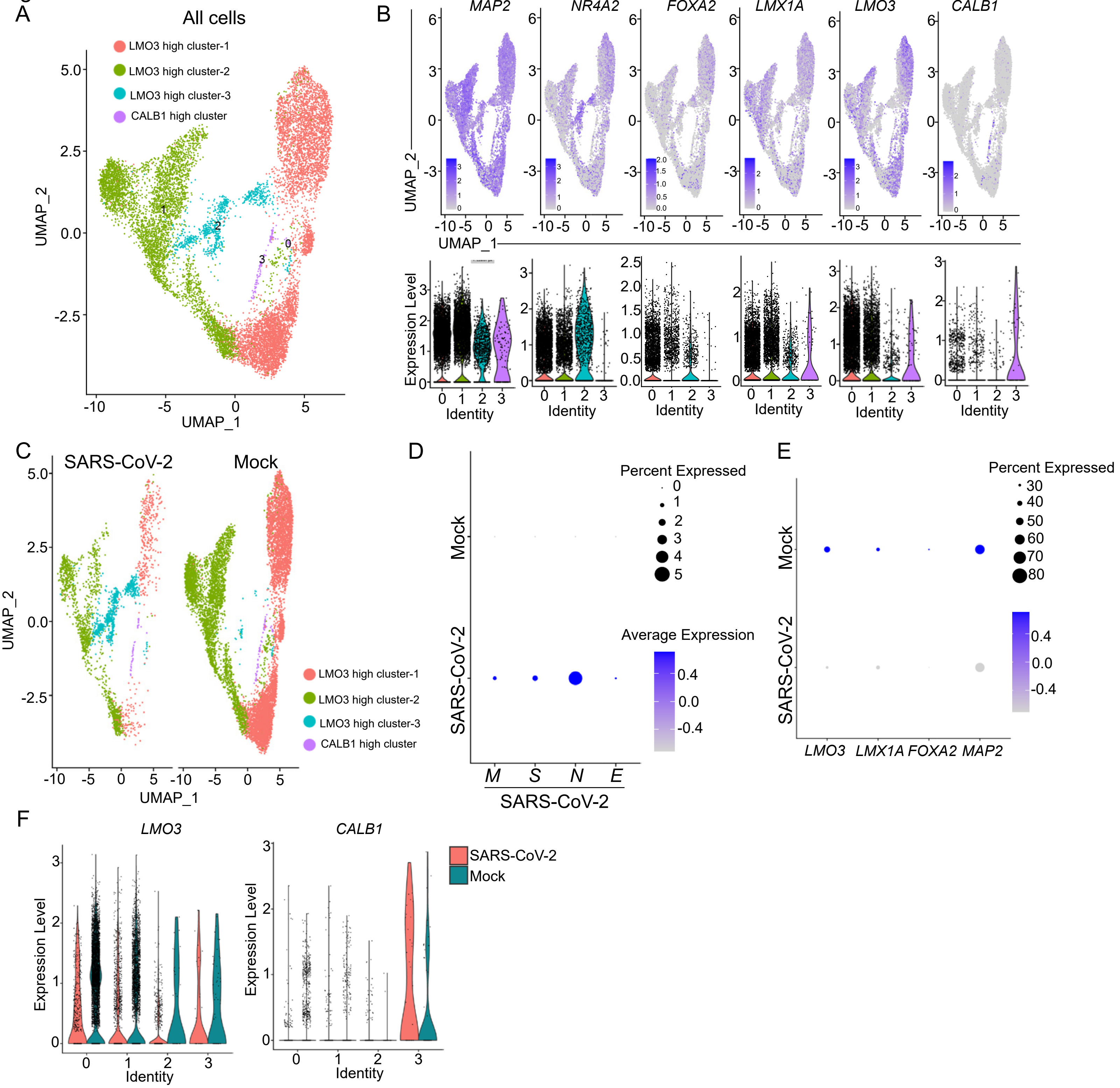
A
All cells
LMO3 high cluster-1
LMO3 high cluster-2
LMO3 high cluster-3
CALB1 high cluster
UMAP_2
UMAP_1
B
MAP2
NR4A2
FOXA2
LMX1A
LMO3
CALB1
Expression Level
Identity
C
SARS-CoV-2
Mock
D
Percent Expressed
Average Expression
M
S
N
E
E
LMX1A
FOXA2
F
Expression Level

Figure S2. hPSC-derived DA neurons are susceptible and permissive to SARS-CoV-2 infection. Related to Figure 2.

(A) UMAP analysis of the mock and SARS-CoV-2 infected purified NURR1: GFP-DA neurons at 48 hpi (MOI=0.2).

(B) UMAP and violin plot analysis of DA neuron marker genes.

(C) UMAP analysis of the mock and SARS-CoV-2 infected purified NURR1: GFP-DA neurons at 48 hpi (MOI=0.2).

(D) Dot plot analysis of SARS-CoV-2 viral transcripts in the mock and SARS-CoV-2 infected purified NURR1: GFP-DA neurons at 48 hpi (MOI=0.2).

(E) Dot plot analysis of expression levels of DA neuron marker genes in the mock and SARS-CoV-2 infected purified NURR1: GFP-DA neurons at 48 hpi (MOI=0.2).

(F) Violin plot analysis of expression levels of DA neuron marker genes in different populations of the mock and SARS-CoV-2 infected purified NURR1: GFP-DA neurons at 48 hpi (MOI=0.2).

Figure S3

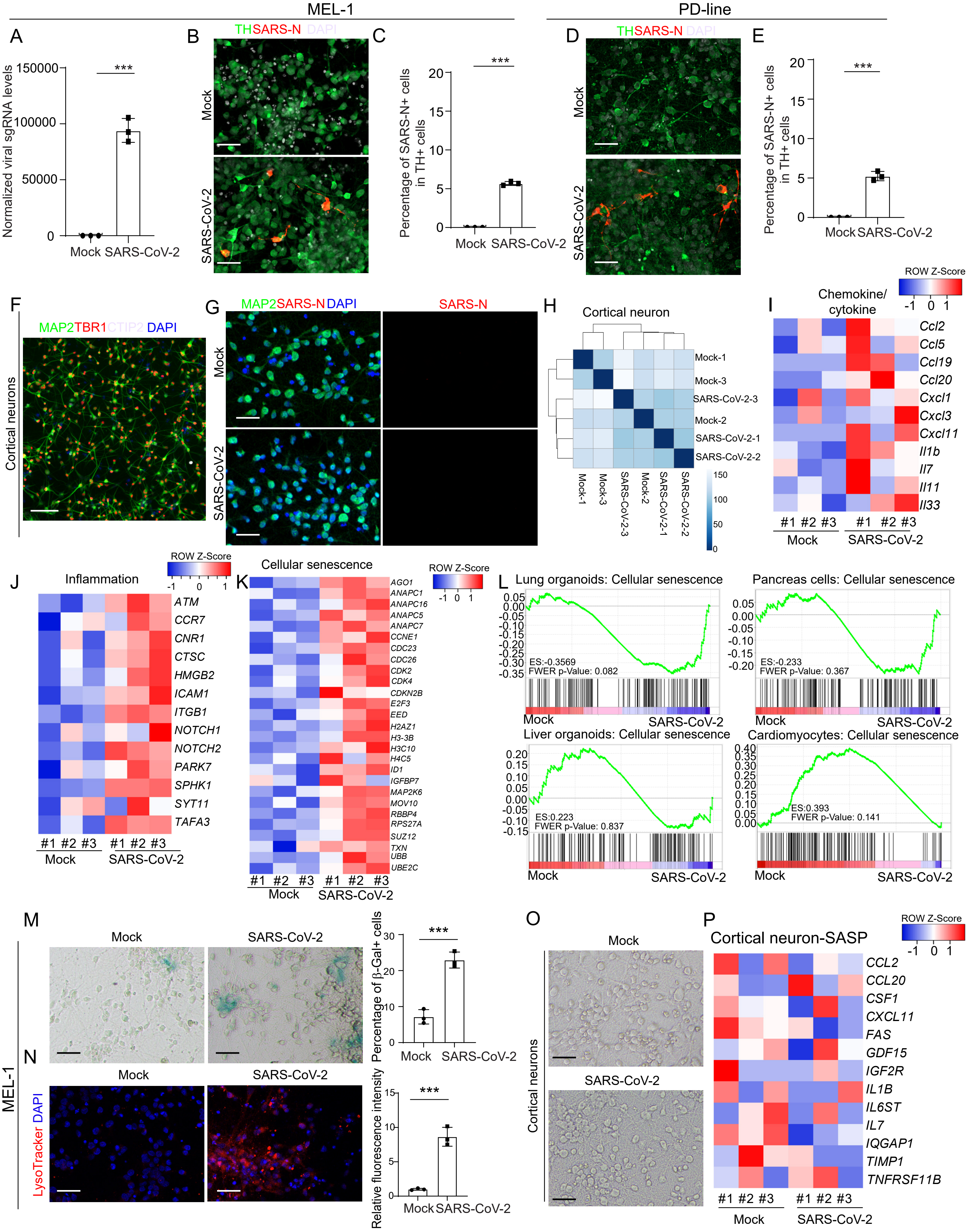

MEL-1
PD-line
A
B
C
D
E
F
G
H
I
J
K
L
M
N
O
P
TH SARS-N DAPI
Mock
SARS-CoV-2
Normalized viral sgRNA levels
Percentage of SARS-N+ cells in TH+ cells
***
Cortical neurons
MAP2 TBR1 CTIP2 DAPI
MAP2 SARS-N DAPI
SARS-N
Cortical neuron
Mock-1
Mock-3
SARS-CoV-2-3
Mock-2
SARS-CoV-2-1
SARS-CoV-2-2
Chemokine/ cytokine
ROW Z-Score
Ccl2
Ccl5
Ccl19
Ccl20
Cxcl1
Cxcl3
Cxcl11
Il1b
Il7
Il11
Il33
#1 #2 #3
Inflammation
ATM
CCR7
CNR1
CTSC
HMGB2
ICAM1
ITGB1
NOTCH1
NOTCH2
PARK7
SPHK1
SYT11
TAFA3
Cellular senescence
AGO1
ANAPC1
ANAPC16
ANAPC5
ANAPC7
CCNE1
CDC23
CDC26
CDK2
CDK4
CDKN2B
E2F3
EED
H2AZ1
H3-3B
H3C10
H4C5
ID1
IGFBP7
MAP2K6
MOV10
RBBP4
RPS27A
SUZ12
TXN
UBB
UBE2C
Lung organoids: Cellular senescence
ES:-0.3569
FWER p-Value: 0.082
Pancreas cells: Cellular senescence
ES:-0.233
FWER p-Value: 0.367
Liver organoids: Cellular senescence
ES:0.223
FWER p-Value: 0.837
Cardiomyocytes: Cellular senescence
ES:0.393
FWER p-Value: 0.141
Percentage of β-Gal+ cells
LysoTracker DAPI
Relative fluorescence intensity
Cortical neuron-SASP
CCL2
CCL20
CSF1
CXCL11
FAS
GDF15
IGF2R
IL1B
IL6ST
IL7
IQGAP1
TIMP1
TNFRSF11B

Figure S3. SARS-CoV-2 infection of hPSC-derived DA neurons triggered senescence phenotype. Related to Figure 1 and Figure 3.

(A) RT-qPCR analysis of total RNA extracted from MEL1-derived DA neurons at 48 hpi of SARS-CoV-2 infection (MOI=0.2) for viral N sgRNA. The graph depicts the mean sgRNA level normalized to ACTB. n=3 independent biological replicates.

(B and C) Representative confocal images (B) and quantification of the percentage of SARS-N$^+$ cells in TH$^+$ cells (C) of MEL1-DA neurons infected with SARS-CoV-2 (MOI=0.1) at 72 hpi using antibodies against SARS-CoV-2 Nucleocapsid protein (SARS-N) and marker for DA neurons. Scale bar=50μm. n=3 independent biological replicates.

(D and E) Representative confocal images (D) and quantification of the percentage of SARS-N$^+$ cells in TH$^+$ cells (E) of SNCA PD line-2copy-DA neurons infected with SARS-CoV-2 (MOI=0.1) at 72 hpi using antibodies against SARS-CoV-2 Nucleocapsid protein (SARS-N) and marker for DA neurons. Scale bar=50 μm. n=3 independent biological replicates. N=3 independent biological replicates.

(F) Representative confocal images of H9-cortical neurons using antibodies against MAP2, TBR1, and CTIP2. Scale bar=100 μm. n=3 independent biological replicates.

(G) Representative confocal images of H9-cortical neurons infected with SARS-CoV-2 (MOI=0.1) at 72 hpi using antibodies against SARS-CoV-2 Nucleocapsid protein (SARS-N) and MAP2. Scale bar=50μm. n=3 independent biological replicates.

(H) Clustering analysis of the mock or SARS-CoV-2 infected H9-derived cortical neurons at 48 hpi (MOI=0.2).

(I and **J)** Heatmap of chemokine/cytokines (I) and inflammation associated genes (J) in the mock or SARS-CoV-2 infected NURR1: GFP H9-derived DA neurons at 48 hpi (MOI=0.2).

(K) Heatmap of senescence associated genes in the mock or SARS-CoV-2 infected NURR1: GFP H9-derived DA neurons at 48 hpi (MOI=0.2).

(L) Gene set enrichment analysis (GSEA) of cellular senescence pathway in the mock or SARS-CoV-2 infected hPSC-derived lung organoids (GSE155241), pancreas cells (GSE151803), liver organoids (GSE151803) and cardiomyocytes (GSE151880).

(M) β-Gal staining and quantification of the mock or SARS-CoV-2 infected MEL1-derived DA neurons at 72 hpi (MOI=0.1). Scale bar=75μm. n=3 independent biological replicates.

(N) Representative confocal images and quantification of MEL1-DA neurons infected with the mock or SARS-CoV-2 (MOI=0.1) at 72 hpi using LysoTracker. Scale bar=50μm. n=3 independent biological replicates.

(O) β-Gal staining and quantification of the mock or SARS-CoV-2 infected H9-derived cortical neurons at 72 hpi (MOI=0.1). Scale bar=75μm. n=3 independent biological replicates.

(P) Heatmap of SASP associated genes in the mock or SARS-CoV-2 infected H9-derived cortical neurons at 48 hpi (MOI=0.2).

Data was presented as mean ± STDEV. *P* values were calculated by unpaired two-tailed Student's t test. *$P < 0.05$, **$P < 0.01$, and ***$P < 0.001$.

Figure S4

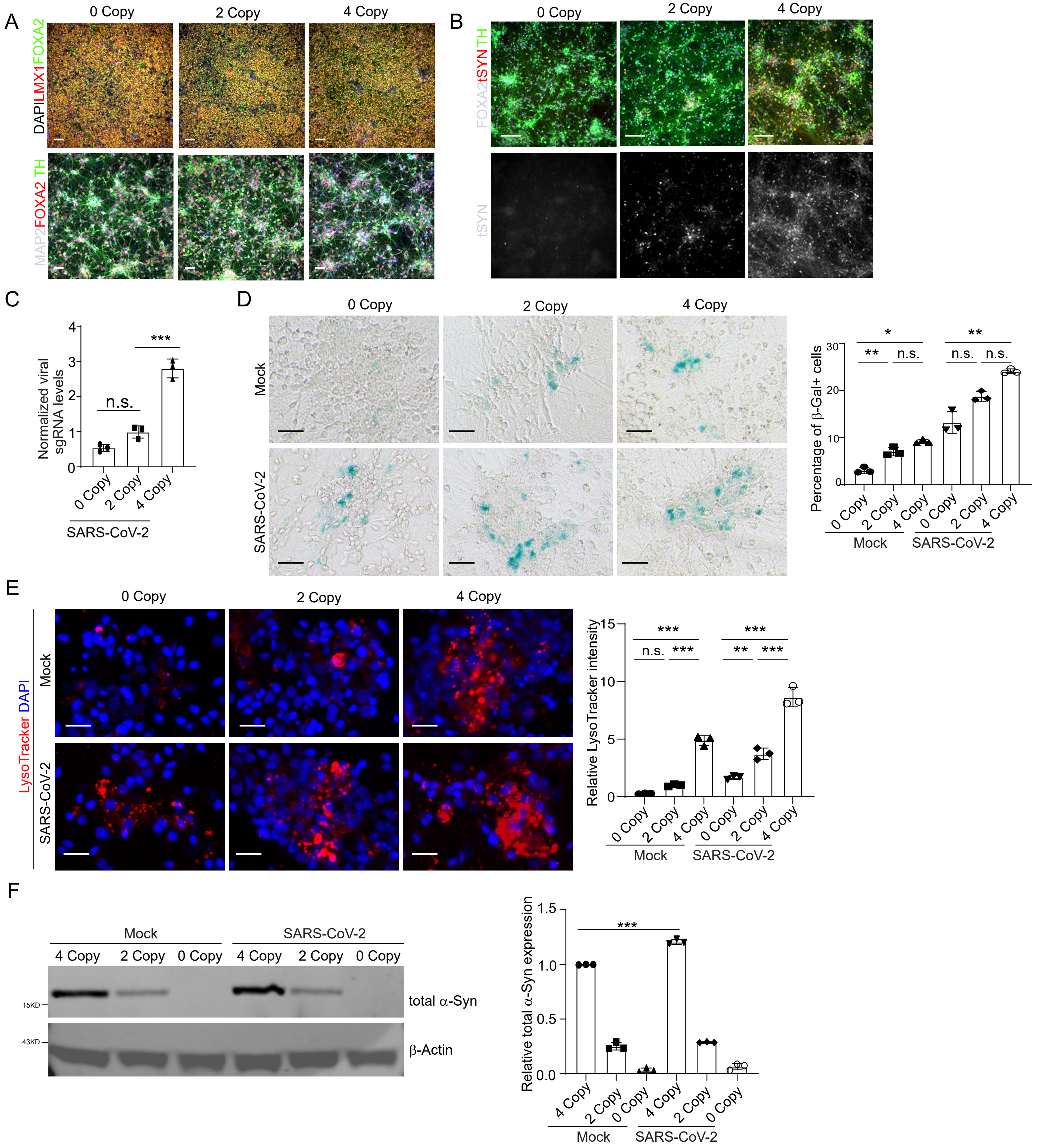
A
0 Copy
2 Copy
4 Copy
DAPILMX1FOXA2
MAP2FOXA2 TH
B
FOXA2tSYNTH
tSYN
C
Normalized viral sgRNA levels
***
n.s.
SARS-CoV-2
D
Mock
SARS-CoV-2
Percentage of β-Gal+ cells
*
**
n.s.
E
LysoTracker DAPI
Relative LysoTracker intensity
***
F
4 Copy
2 Copy
0 Copy
15KD
43KD
total α-Syn
β-Actin
Relative total α-Syn expression

Figure S4. SARS-CoV-2 infection on SNCA 0 copy, 2 copy and 4 copy PD-iPSC-derived DA neurons. Related to Figure 3.

(A) Representative confocal images of *SNCA* lines-derived DA neurons at day 20 (upper) and day 45 (lower) using antibodies against markers for DA neurons. Scale bar=50 μm. n=3 independent biological replicates.

(B) Representative confocal images of *SNCA* lines-derived DA neurons at day 45 using antibodies against total a-Syn and markers for DA neurons. Scale bar=50 μm. n=3 independent biological replicates.

(C) qRT-PCR analysis of total RNA extracted from *SNCA* lines-derived DA neurons at 48 hpi of SARS-CoV-2 infection (MOI=0.2) for viral N sgRNA. The graph depicts the mean sgRNA level normalized to *ACTB*. n=3 independent biological replicates.

(D) β-Gal staining and quantification of the mock or SARS-CoV-2 infected *SNCA* lines-derived DA neurons at 72 hpi (MOI=0.1). Scale bar=75μm. n=3 independent biological replicates.

(E) Representative confocal images and quantification of *SNCA* lines-derived DA neurons infected with the mock or SARS-CoV-2 (MOI=0.1) at 72 hpi stained with LysoTracker. Scale bar=50 μm. n=3 independent biological replicates.

(F) Western blot analysis and quantification of senescence related proteins of total α-Syn in mock or SARS-CoV-2 infected *SNCA* lines-derived DA neurons at 48 hpi (MOI=0.2). n=3 independent biological replicates.

Data was presented as mean ± STDEV. *P* values were calculated by unpaired two-tailed Student's t test. n.s. no significance, *$P < 0.05$, **$P < 0.01$, and ***$P < 0.001$.

Figure S5

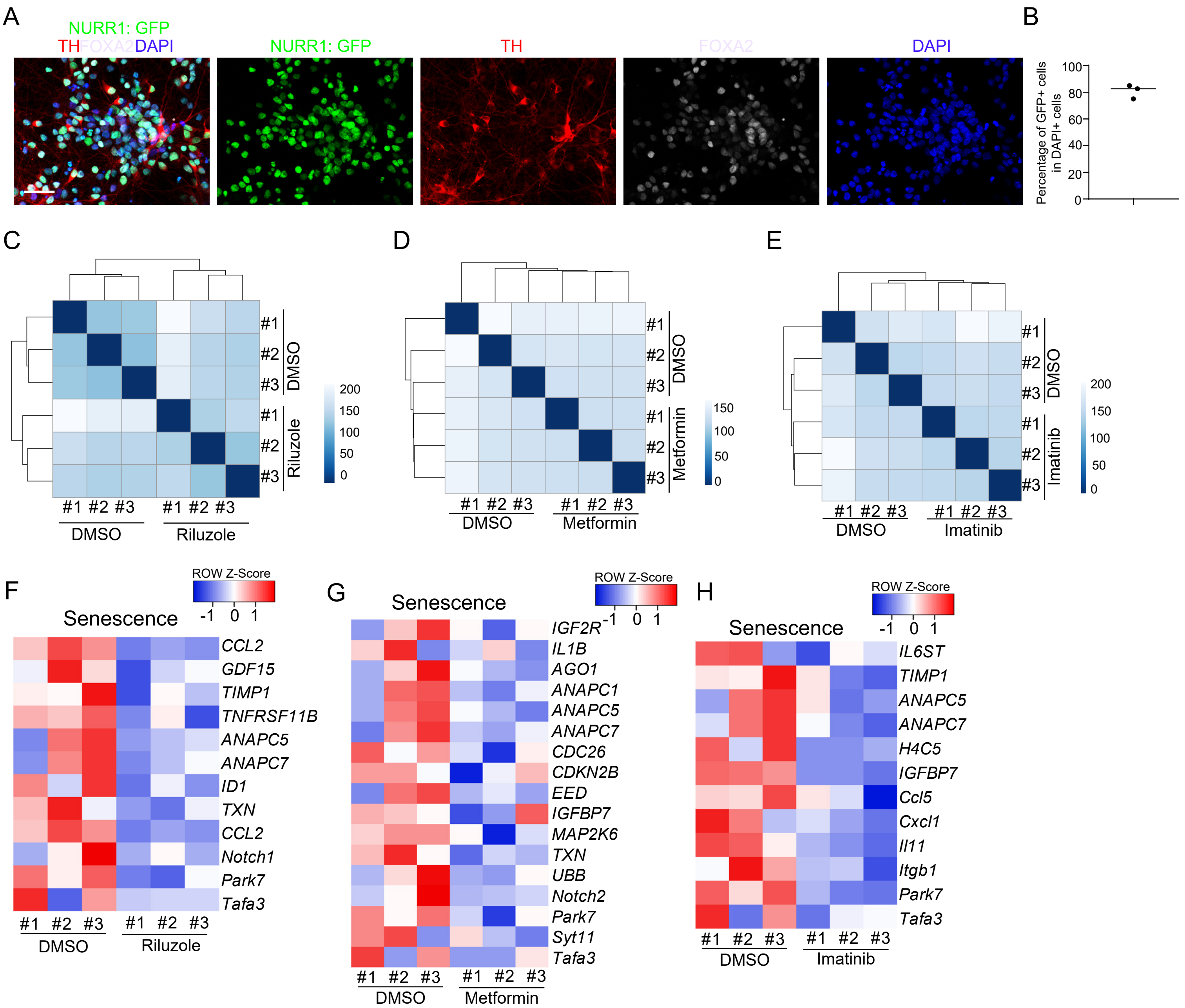
A
NURR1: GFP
TH FOXA2 DAPI
NURR1: GFP
TH
FOXA2
DAPI
B
Percentage of GFP+ cells in DAPI+ cells
100
80
60
40
20
0
C
#1
#2
#3
DMSO
#1
#2
#3
Riluzole
200
150
100
50
0
#1 #2 #3
DMSO
#1 #2 #3
Riluzole
D
DMSO
Metformin
150
100
50
0
E
DMSO
Imatinib
200
150
100
50
0
F
ROW Z-Score
-1 0 1
Senescence
CCL2
GDF15
TIMP1
TNFRSF11B
ANAPC5
ANAPC7
ID1
TXN
CCL2
Notch1
Park7
Tafa3
DMSO
Riluzole
G
Senescence
IGF2R
IL1B
AGO1
ANAPC1
ANAPC5
ANAPC7
CDC26
CDKN2B
EED
IGFBP7
MAP2K6
TXN
UBB
Notch2
Park7
Syt11
Tafa3
DMSO
Metformin
H
Senescence
IL6ST
TIMP1
ANAPC5
ANAPC7
H4C5
IGFBP7
Ccl5
Cxcl1
Il11
Itgb1
Park7
Tafa3
DMSO
Imatinib

Figure S5. Three drug candidates rescue senescence phenotype of SARS-CoV-2 infected hPSC-derived DA neurons. Related to Figure 4.

(A and B) Representative confocal images (A) and quantification of the percentage of NURR1-GFP$^+$ cells (B) of NURR1: GFP H9 lines-derived DA neurons for drug screening. Scale bar=50 µm. n=3 independent biological replicates. Data was presented as mean ± STDEV.

(C-E) Clustering analysis of gene expression profiles of DMSO or drug candidate-treated purified hPSC-derived DA neurons at 48 hpi upon SARS-CoV-2 infection (MOI=0.1).

(F-H) Heatmap of SASP associated genes of DMSO or drug candidate-treated purified hPSC-derived DA neurons at 48 hpi upon SARS-CoV-2 infection (MOI=0.1).

Figure S6

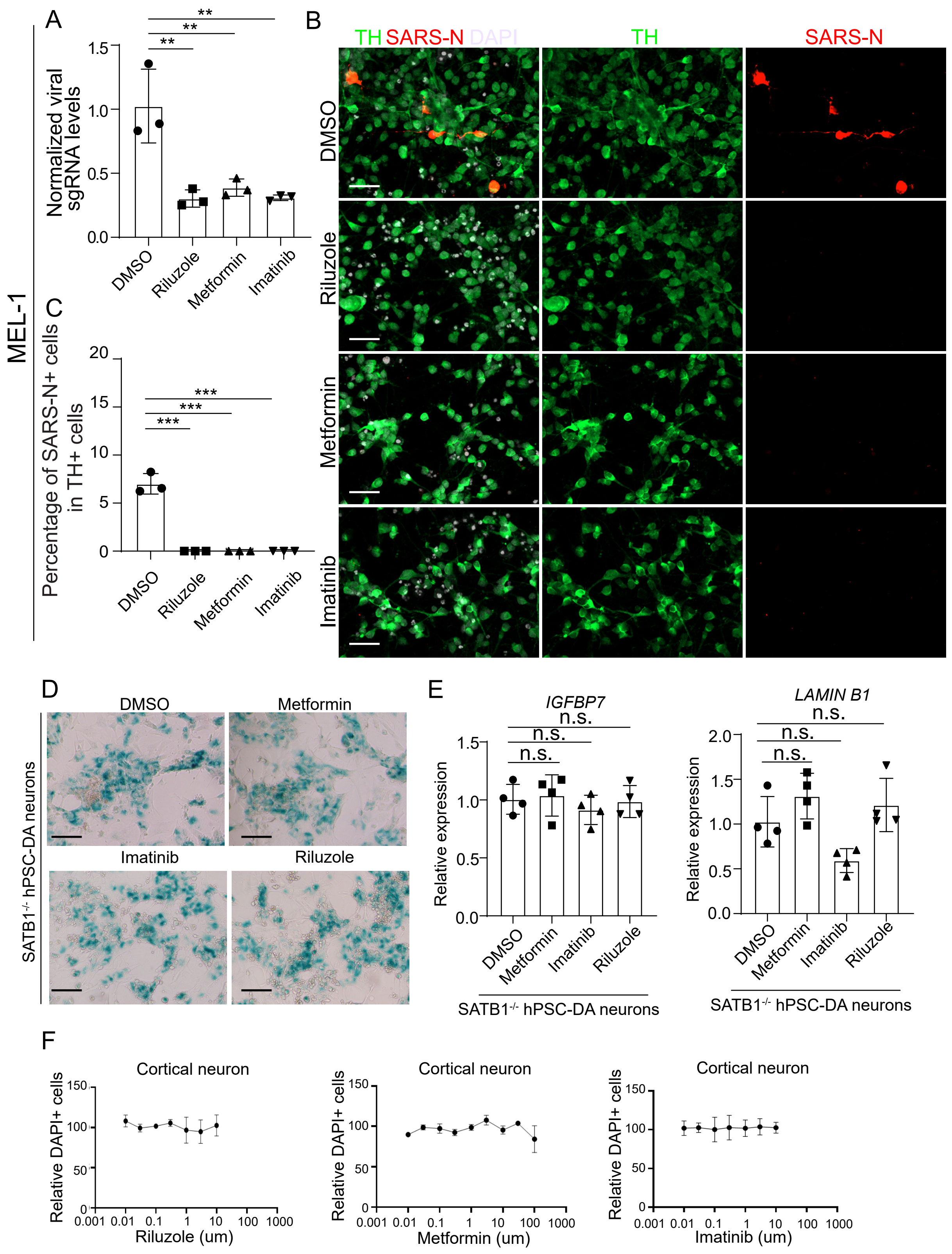

A
B
C
D
E
F
MEL-1
Normalized viral sgRNA levels
1.5
1.0
0.5
0.0
**
DMSO
Riluzole
Metformin
Imatinib
TH SARS-N DAPI
TH
SARS-N
Percentage of SARS-N+ cells in TH+ cells
20
15
10
5
0
***
SATB1-/- hPSC-DA neurons
IGFBP7
LAMIN B1
n.s.
Relative expression
2.0
Cortical neuron
Relative DAPI+ cells
150
100
50
0.001 0.01 0.1 1 10 100 1000
Riluzole (um)
Metformin (um)
Imatinib (um)

Figure S6. Three drug candidates block SARS-CoV-2 infection of hPSC-derived DA neurons. Related to Figure 5 and Figure 6.

(A) RT-qPCR analysis of total RNA extracted from DMSO or drug candidates-pre-treated MEL1-derived DA neurons following SARS-CoV-2 infection (MOI=0.2) for viral N sgRNA at 48 hpi. The graph depicts the mean sgRNA level normalized to *ACTB*. n=3 independent biological replicates.

(B and C) Representative confocal images (B) and quantification of the percentage of SARS-N^+ cells in TH^+ cells (C) of DMSO or drug candidates-pre-treated MEL1-DA neurons following SARS-CoV-2 infection (MOI=0.2) at 72 hpi using antibodies against SARS-CoV-2 Nucleocapsid protein (SARS-N) and marker for DA neurons. Scale bar=50 μm. n=3 independent biological replicates.

(D) β-Gal staining and quantification of DMSO or drug candidates-treated *SATB1*$^{-/-}$ hPSC-derived DA neurons at 72 hpi (MOI=0.1). Scale bar=75μm. n=3 independent biological replicates.

(E) RT-qPCR analysis of senescence related genes of *IGFBP7* and *LAMIN B1* in DMSO or drug candidates-treated *SATB1*$^{-/-}$ hPSC-derived DA neurons at 48 hpi (MOI=0.2). n=3 independent biological replicates.

(F) Cytotoxicity curves of riluzole, metformin and imatinib in H9-derived cortical neurons. n=3 independent biological replicates.

Data was presented as mean ± STDEV. *P* values were calculated by unpaired two-tailed Student's t test. n.s. no significance, $^{**}P < 0.01$, and $^{***}P < 0.001$.

Figure S7

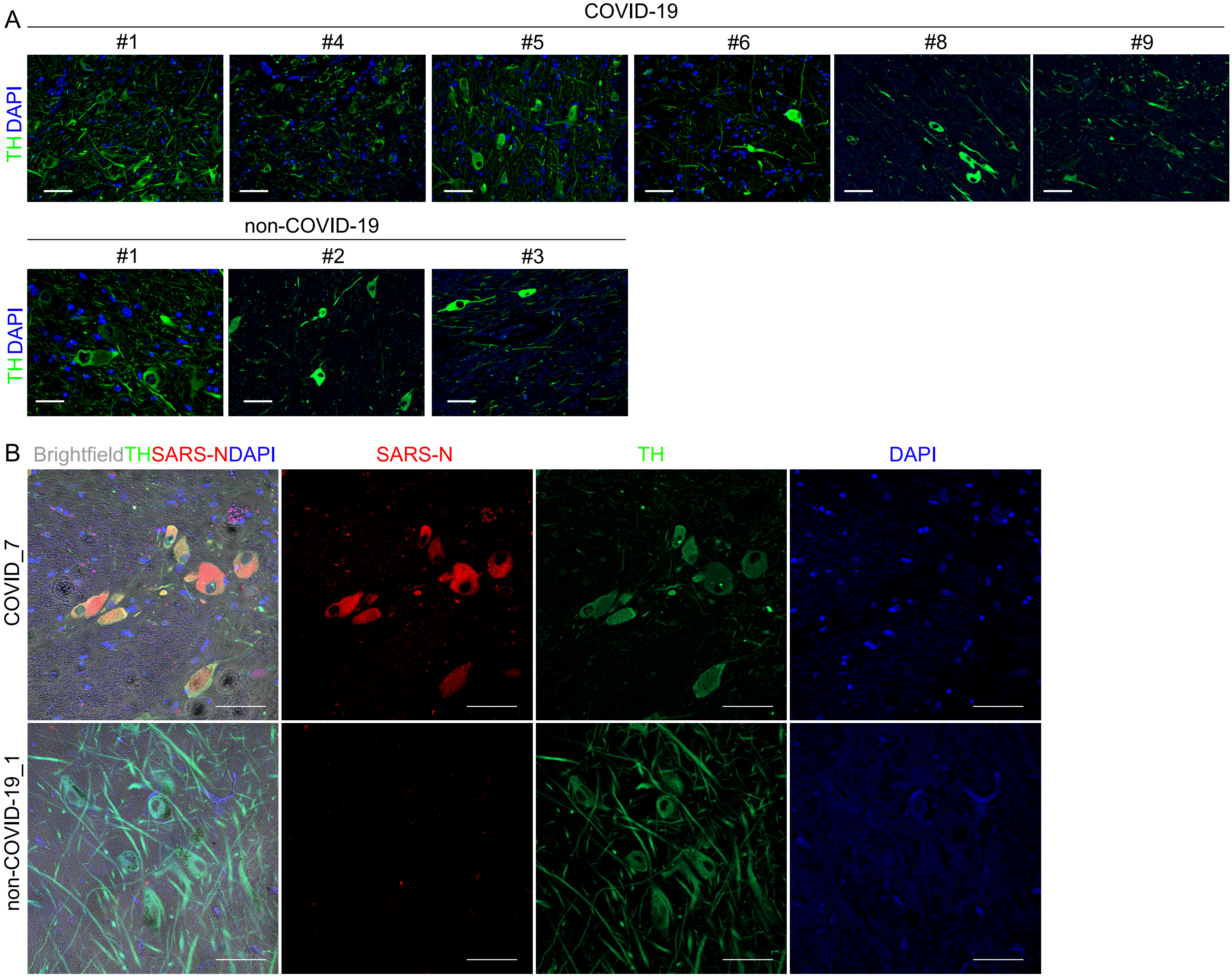
A
COVID-19
#1
#4
#5
#6
#8
#9
TH DAPI
non-COVID-19
#1
#2
#3
TH DAPI
B
BrightfieldTHSARS-NDAPI
SARS-N
TH
DAPI
COVID_7
non-COVID-19_1

Figure S7. Inflammatory and senescence phenotypes were detected in autopsy substantia nigra samples of COVID-19 patients. Related to Figure 7.

(A) Representative confocal images of TH staining in the autopsy substantia nigra sections of COVID-19 patients versus non-COVID-19 patients. (Cohort 1: N=6 COVID-19 patients; N=3 non-COVID-19 patients). Scale bar=50µm.

(B) Representative confocal images of SARS-N and TH staining in the autopsy substantia nigra sections of COVID-19 patient versus non-COVID-19 patient. (Cohort 2: N=1 COVID-19 patients; N=1 non-COVID-19 patients). Scale bar=50µm.

Table S1. Demographics and pathological characterization of control autopsy cohort. Related to Figure 7 and Figure S7.

Table S2. Demographics and pathological characterization of COVID-19 autopsy cohort. Related to Figure 7 and Figure S7.

Table S3. Demographics and pathological characterization of PD(D) autopsy cohort. Related to Figure 7 and Figure S7.

Table S4. Patient information. Related to Figure 7 and Figure S7.

	**Patient ID**	**Gender**	**Age**	**Experiments**	**Source**
Cohort 1	COVID_1	Male	66	Fig. 7A-7E and Fig. S7A	Columbia University
	COVID_4	Female	78	Fig. 7A-7E and Fig. S7A	Columbia University
	COVID_5	Female	80	Fig. 7A-7E and Fig. S7A	Columbia University
	COVID_6	Male	80	Fig. 7A-7E and Fig. S7A	Columbia University
	COVID_7	Male	74	Fig. S7B	The Netherlands
	COVID_8	Male	67	Fig. 7A-7E, Fig. 7F and Fig. S7A	The Netherlands
	COVID_9	Male	59	Fig. 7A-7E, Fig. 7F and Fig. S7A	The Netherlands
	non-COVID_1	Male	72	Fig. 7A-7E, Fig. 7F and Fig. S7A	The Netherlands
	non-COVID_2	Male	61	Fig. 7A-7E, Fig. 7F and Fig. S7A	The Netherlands
	non-COVID_3	Female	61	Fig. 7A-7E and Fig. S7A	Columbia University
	non-COVID_4	Male	82	Fig. 7F	The Netherlands
Cohort 2	-	-	-	Fig. 7G-7H and Table S1-S3	The Netherlands

Table S5. Antibodies used for immunocytochemistry, intracellular flow cytometry analysis and western blotting analysis. Related to STAR Methods.

Usage	Antibody	Clone #	Host	Catalog #	Vendor	Dilution
Immunocytochemistry	Anti-SARS-CoV/SARS-CoV-2 Nucleocapsid	001	Rabbit	40143-R001	Sino Biological	1:500
Immunocytochemistry	ACE2	Polyclonal	Rabbit	ab15348	Abcam	1:500
Immunocytochemistry	Firefly luciferase Monoclonal Antibody (CS 17)	CS 17	Mouse	35-6700	Thermo Fisher Scientific	1:200
Immunocytochemistry and western blot	Anti-Alpha Synuclein	Syn211	Mouse	S5566-100UL	Millipore	1:250
Immunocytochemistry	Anti-MAP2 antibody	Polyclonal	Chicken	ab5392	Abcam	1:5000
Western blot	p21 Waf1/Cip1 (12D1) Rabbit mAb	12D1	Rabbit	2947	Cell Signaling	1:500
Western blot	Human Lamin B1 Antibody	919007	Mouse	MAB8525	R&D Systems	1:250
Immunocytochemistry	anti-α-Synuclein Phospho	P-syn/81A	Mouse	MMS-5091	Biolegend	1:1000
Immunocytochemistry	Goat polyclonal anti-FOXA2	Polyclonal	Goat	AF2400	R&D Systems	1:250
Immunocytochemistry	Recombinant Anti-TBR1 antibody	EPR8138 (2)	Rabbit	ab183032	Abcam	1:100
Immunocytochemistry	Anti-Ctip2 antibody	25B6	Rat	ab18465	Abcam	1:500
Immunocytochemistry	Anti-Tyrosine Hydroxylase antibody - Neuronal Marker	Polyclonal	Rabbit	ab112	Abcam	1:500

Immunocytochemistry	Human/Mouse Tyrosine Hydroxylase Antibody	779427	Mouse	MAB7566	R&D Systems	1:200
Immunocytochemistry	Anti-FOXA2 Antibody	M-20	Goat	sc-6554	Santa Cruz	1:150
Immunocytochemistry	Anti-GFP antibody	Polyclonal	Chicken	ab13970	Abcam	1:1000
Immunocytochemistry	Rabbit polyclonal anti-LMX-1	Polyclonal	Rabbit	AB10533	Millipore	1:1000
Immunocytochemistry	Donkey anti-Mouse IgG (H+L) Cross-Adsorbed Secondary Antibody, Alexa Fluor 488	Polyclonal	Donkey	A-21202	Thermo Fisher Scientific	1:500
Immunocytochemistry	Donkey anti-Rabbit IgG (H+L) Secondary Antibody, Alexa Fluor 594	Polyclonal	Donkey	A-21207	Thermo Fisher Scientific	1:500
Immunocytochemistry	Donkey anti-Mouse IgG (H+L) Secondary Antibody, Alexa Fluor 594 conjugate	Polyclonal	Donkey	A-21203	Thermo Fisher Scientific	1:500
Immunocytochemistry	Donkey anti-Mouse IgG (H+L) Secondary Antibody, Alexa Fluor 647	Polyclonal	Donkey	A-31571	Thermo Fisher Scientific	1:500
Immunocytochemistry	Alexa Fluor 488 AffiniPure Donkey Anti-Chicken IgY (IgG) (H+L)	Polyclonal	Donkey	703-545-155	Jackson Immunoresearch Labs	1:500
Immunocytochemistry	Donkey anti-Goat IgG (H+L) Cross-Adsorbed	Polyclonal	Donkey	A-21447	Thermo Fisher	1:500

	Secondary Antibody, Alexa Fluor 647				Scientific	
Immunocytochemistry	Donkey anti-Goat IgG Secondary Antibody, Alexa Fluor 555	Polyclonal	Donkey	A32816	Thermo Fisher	1:500
Immunocytochemistry	Donkey anti-Rabbit IgG Secondary Antibody, Alexa Fluor 647	Polyclonal	Donkey	A32795	Thermo Fisher	1:500

Table S6. Primers used for RT-qPCR. Related to STAR Methods.

Primer name	Sequence
ACTB-Forward	*CGTCACCAACTGGGACGACA*
ACTB-Reverse	*CTTCTCGCGGTTGGCCTTGG*
SARS-CoV-2-TRS-L	*CTCTTGTAGATCTGTTCTCTAAACGAAC*
SARS-CoV-2-TRS-N	*GGTCCACCAAACGTAATGCG*
LaminB1-F	*AAGCATGAAACGCGCTTGG*
LaminB1-R	*AGTTTGGCATGGTAAGTCTGC*
IGFBP7-F	*ATCCCGACACCTGTCCTCAT*
IGFBP7-R	*CCCAGCCAGTTACTTCATGCT*

Reference

1. Riessland, M., Kolisnyk, B., Kim, T.W., Cheng, J., Ni, J., Pearson, J.A., Park, E.J., Dam, K., Acehan, D., Ramos-Espiritu, L.S., et al. (2019). Loss of SATB1 Induces p21-Dependent Cellular Senescence in Post-mitotic Dopaminergic Neurons. Cell Stem Cell *25*, 514-530 e518. 10.1016/j.stem.2019.08.013.
